# Structural basis of carnitine monooxygenase CntA substrate specificity, inhibition, and intersubunit electron transfer

**DOI:** 10.1074/jbc.RA120.016019

**Published:** 2020-11-23

**Authors:** Mussa Quareshy, Muralidharan Shanmugam, Eleanor Townsend, Eleanor Jameson, Timothy D.H. Bugg, Alexander D. Cameron, Yin Chen

**Affiliations:** 1 School of Life Sciences, University of Warwick, Coventry, UK; 2 Manchester Institute of Biotechnology & Photon Science Institute, The University of Manchester, Manchester, UK; 3 Department of Chemistry, University of Warwick, Coventry, UK

**Keywords:** gut microbiota, carnitine oxygenase, CntA, EPR, inhibitor, EPR, electron paramagnetic resonance, NO, nitric oxide, TMA, trimethylamine

## Abstract

Microbial metabolism of carnitine to trimethylamine (TMA) in the gut can accelerate atherosclerosis and heart disease, and these TMA-producing enzymes are therefore important drug targets. Here, we report the first structures of the carnitine oxygenase CntA, an enzyme of the Rieske oxygenase family. CntA exists in a head-to-tail α_3_ trimeric structure. The two functional domains (the Rieske and the catalytic mononuclear iron domains) are located >40 Å apart in the same monomer but adjacent in two neighboring monomers. Structural determination of CntA and subsequent electron paramagnetic resonance measurements uncover the molecular basis of the so-called bridging glutamate (E205) residue in intersubunit electron transfer. The structures of the substrate-bound CntA help to define the substrate pocket. Importantly, a tyrosine residue (Y203) is essential for ligand recognition through a π-cation interaction with the quaternary ammonium group. This interaction between an aromatic residue and quaternary amine substrates allows us to delineate a subgroup of Rieske oxygenases (group V) from the prototype ring-hydroxylating Rieske oxygenases involved in bioremediation of aromatic pollutants in the environment. Furthermore, we report the discovery of the first known CntA inhibitors and solve the structure of CntA in complex with the inhibitor, demonstrating the pivotal role of Y203 through a π–π stacking interaction with the inhibitor. Our study provides the structural and molecular basis for future discovery of drugs targeting this TMA-producing enzyme in human gut.

The vast array of cohabiting microorganisms in the human gut impose a discernible influence on human well-being and disease states. There is a considerable interest in the past decade to investigate the formation of methylated amines in human health, particularly with regard to cardiovascular diseases and nonalcoholic fatty liver diseases (1, 2, 3, 4, 5). Dietary intake of quaternary amines such as choline and carnitine, both of which are prevalent in the human diet, can be processed by gut microbiota to produce small methylated amines (*e.g.*, trimethylamine, TMA), which enter vascular circulation leading to subsequent hepatic oxidation to trimethylamine oxide (TMAO). TMAO is linked to cardiovascular disease, kidney disease, diabetes, and various forms of cancers (6, 7, 8). These TMA-producing and metabolizing enzymes from gut microbiota therefore represent promising new drug targets (9, 10, 11).

The key microbial enzymes responsible for TMA formation from choline and carnitine have only been identified relatively recently (12, 13, 14). Choline-TMA lyases belong to a large family of proteins, attacking the carbon–nitrogen (C-N) bond in choline using a radical species generated from a conserved glycine residue. The structure of choline-TMA lyase CutC has been solved recently (15, 16), aiding the development of inhibitors (substrate analogs) for attenuating TMA formation from choline (17, 18, 19, 20). The carnitine monooxygenase (CntA) and associated reductase (CntB) responsible for carnitine oxidation to TMA (Fig. 1*A*) was originally identified in *Acinetobacter* spp. but was subsequently found to be present in a range of gut microbiota species (13, 14, 21). CntA belongs to a large group of non-heme-iron–containing Rieske oxygenase family (13). Rieske oxygenases are typically multicomponent enzyme systems involving an oxygenase, a reductase, and sometimes a separate flavin cofactor (22). The oxygenase component has a conserved [2Fe-2S] Rieske center coordinated by two cysteine and two histidine residues and a catalytic mononuclear iron (Fe) center (23, 24). A reductase component reduces pyridine nucleotides, generating electrons, which are ultimately transferred to the Rieske oxygenase for substrate oxidation. The archetypal Rieske oxygenases, also known as ring-hydroxylating Rieske oxygenases, catalyze the oxidation of a range of aromatic and polyaromatic substrates; as such, they are important in bioremediation of environmental pollutants (24, 25). Structural determination of several ring-hydroxylating Rieske oxygenases (*e.g.*, naphthalene dioxygenase and biphenyl dioxygenase) reveals that the Rieske center and the catalytic mononuclear Fe center are usually far apart in the same subunit (>40 Å) and effective electron transfer can only occur across the interface of two neighboring subunits (23, 26, 27).

**Figure 1:**
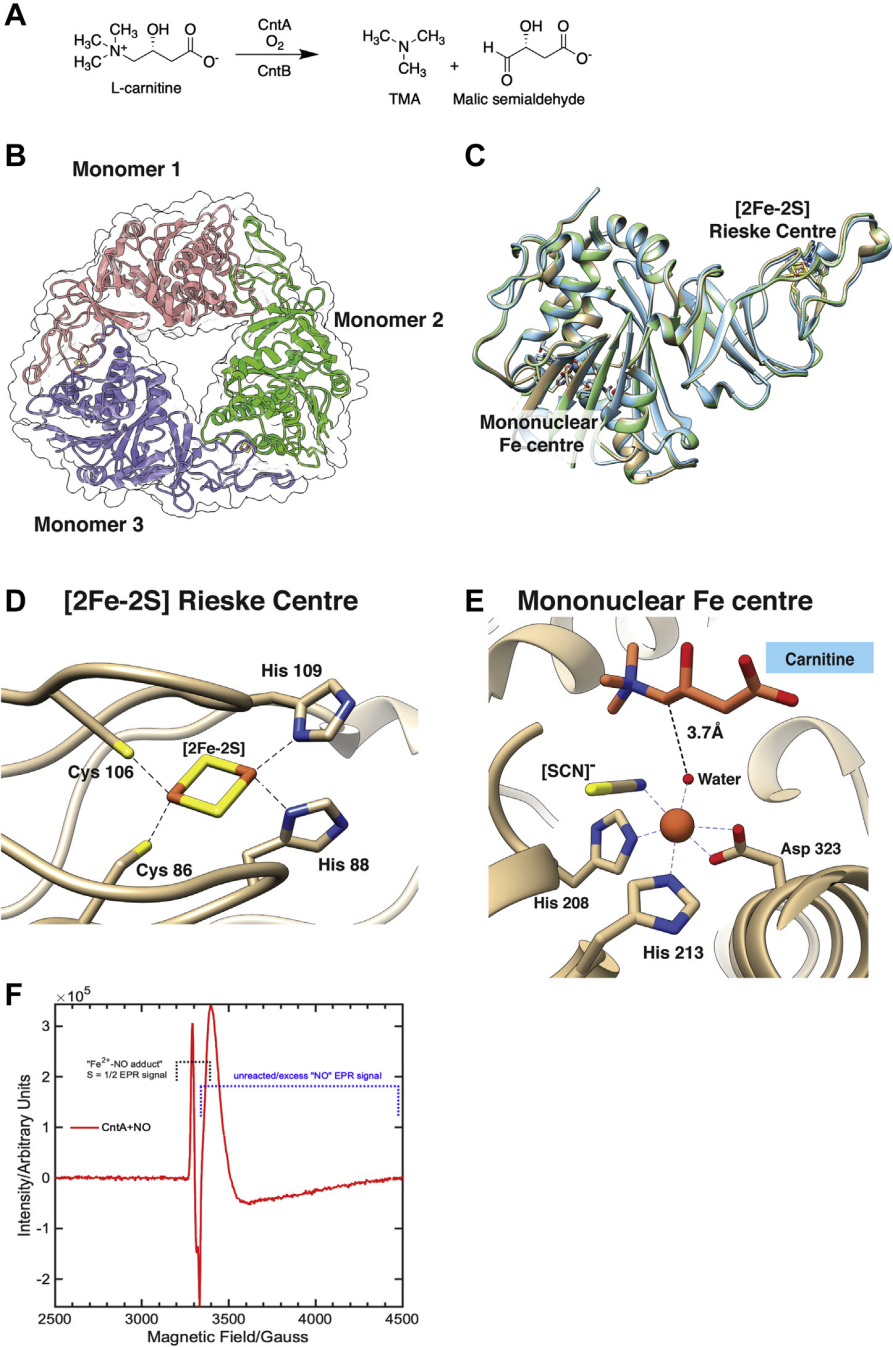
**The overall structure of CntA and its catalytic center.***A*, the reaction of carnitine oxidation by CntAB to trimethylamine (TMA) and malic semialdehyde. *B*, CntA depicted as a homotrimer with the secondary structure represented in a ribbon depiction in a translucent overall surface representation with each monomer in *blue*, *red*, and *green*, respectively. *C*, an overlap of three monomer structure units (apo, carnitine bound, gBB bound), showing no major changes to the tertiary structure of CntA with and without ligands bound. The C and N labels refer to the sequence termini. The Rieske center and the mononuclear Fe center are 44 Å apart in the same subunit. *D*, the Rieske center in CntA is coordinated by Cys86, Cys106, His88, and His109. *E*, the catalytic mononuclear Fe center in CntA is coordinated by a His-His-Asp catalytic triad (His208, His213, Asp323), a water, and thiocyanate ion. Carnitine is shown above the mononuclear Fe center with a distance from the water molecule to the site of substrate cleavage. *F*, electron paramagnetic resonance (EPR) spectrum of as-isolated CntA in the presence of nitric oxide (NO), showing the EPR-active, *S* = ½ species due to the formation of Fe^2+^-NO adduct. EPR conditions: microwave power 30 dB, modulation amplitude 5 G, time constant 81 ms, conversion time 41 ms, sweep time 84 s, receiver gain 60 dB, microwave frequency 9.384 GHz, temperature 20 K.

It is becoming increasingly clear that quaternary amines-oxidizing Rieske oxygenases represent a new emerging clade of the Rieske oxygenases family that is distinct from the archetypal ring-hydroxylating aromatic Rieske oxygenases (13, 27, 28, 29). The carnitine-degrading CntA enzyme is a promising new drug target, given that gut microbial metabolism of carnitine is known to increase plasma TMAO, leading to the subsequent development of atherosclerosis and cardiovascular diseases (9). However, no structure of CntA or description of possible inhibitors has been published. In this study, we report high-resolution crystal structures of CntA with and without substrates bound and report the first known inhibitors for this enzyme and the crystal structure of an inhibitor-bound CntA. Structure, biochemical, and electron paramagnetic resonance (EPR) characterization of CntA and mutants provides novel insights into the mode of inhibition and the inter-/intrasubunit electron transfer in carnitine oxidation. Our work therefore provides the structural and molecular basis for future discovery of drugs targeting this TMA-producing enzyme in human gut.

## Results

### Structure determination of CntA

We set out to solve the structure of CntA from *Acinetobacter baumannii* and successfully obtained structures of CntA in the apo protein form (2.1 Å, PDB 6Y8J) and ligand-bound forms with two substrates, carnitine (2.0 Å, PDB 6Y9D) and γ-butyrobetaine (gBB; 1.6 Å, PDB 6Y8S) (Table 1). We observed a head-to-tail α_3_ trimeric structure for CntA (Fig. 1*B*), with the Rieske [2Fe-2S] cluster and mononuclear Fe center at opposing regions of the monomer 44 Å apart (Fig. 1*C*). The observed assembly of α_3_ homotrimer is in agreement with the analyses by native protein gel (13) and analytic gel filtration (Fig. S1). The two CntA substrate bound structures and apo form are superimposable with an average root-mean-square deviation of 0.487 Å (Table S1).

**Table 1:** X-ray data collection and refinement statistics

Name	CntA apo	CntA + carnitine	CntA + gBB	CntA+MMV12
PDB code	6Y8J	6Y9D	6Y8S	6ZGP
Wavelength (Å)	1.7	1.3	1.3	0.9
Resolution range (Å)	40.1–2.05 (2.12–2.05)	81.42–1.97 (2.04–1.97)	39.78–1.63 (1.69–1.63)	78.64–2.01 (2.08–2.01)
Space group	P 63	P 1 21 1	P 63	P 1 21 1
Unit cell	91.6 91.6 82.96 90 90 120	91.59 177.77 158.8 90 90.17 90	91.16 91.16 81.47 90 90 120	91.07 173.77 157.29 90 90.15 90
Total reflections	224,999 (13,603)	1,180,725 (105,398)	203,443 (9804)	2,229,436 (218,489)
Unique reflections	24,714 (2443)	353,116 (35,196)	47,465 (4434)	322,879 (32,095)
Multiplicity	9.1 (5.6)	3.3 (3.0)	4.3 (2.2)	6.9 (6.8)
Completeness (%)	99.3 (99.2)	98.8 (98.4)	98.8 (93.4)	99.4 (99.1)
Mean I/sigma(I)	7.41 (1.14)	5.2 (1.21)	6.8 (1.13)	6.9 (1.13)
Wilson B-factor (Å^2^)	38	31	24	33
R-merge	0.189 (1.429)	0.149 (0.958)	0.134 (0.755)	0.165 (1.783)
R-meas	0.200 (1.575)	0.178 (1.168)	0.152 (0.967)	0.179 (1.929)
R-pim	0.064 (0.651)	0.096 (0.660)	0.070 (0.595)	0.068 (0.733)
CC_1/2_	0.994 (0.438)	0.984 (0.638)	0.982 (0.517)	0.996 (0.698)
CC∗	0.999 (0.781)	0.996 (0.883)	0.996 (0.825)	0.999 (0.907)
Reflections used in refinement	24,704 (2441)	352,719 (35,160)	47,464 (4434)	321,994 (31,973)
Reflections used for R-free	1223 (101)	17,582 (1828)	2374 (221)	16,243 (1504)
R-work	0.201 (0.429)	0.207 (0.338)	0.1704 (0.323)	0.216 (0.386)
R-free	0.248 (0.530)	0.243 (0.371)	0.193 (0.339)	0.254 (0.421)
CC(work)	0.958 (0.737)	0.961 (0.805)	0.963 (0.726)	0.961 (0.799)
CC(free)	0.942 (0.821)	0.953 (0.760)	0.911 (0.768)	0.948 (0.736)
Number of non-hydrogen atoms	2874	35,752	3117	36,701
macromolecules	2803	34,344	2832	34,901
ligands	4	408	31	588
solvent	67	1000	254	1212
Protein residues	345	4224	350	4328
RMS(bonds) (Å)	0.008	0.009	0.007	0.009
RMS(angles) (°)	1.18	1.10	1.05	1.32
Ramachandran favored (%)	94.67	95.35	95.91	94.76
Ramachandran allowed (%)	5.33	4.50	3.80	4.98
Ramachandran outliers (%)	0.00	0.14	0.29	0.26
Rotamer outliers (%)	0.00	0.87	0.00	0.85
Clashscore	5.86	9.36	3.41	13.20
Average B-factor (Å^2^)	55.95	45.44	35.71	51.59
Macromolecules (Å^2^)	56.24	45.40	34.94	51.60
[2Fe-2S] Rieske Center	44.74	41.35	25.56	48.08
Fe	N/A	45.15	176.41	56.77
Substrate	N/A	47.02	57.75	N/A
Inhibitor	N/A	N/A	N/A	69.75
Solvent (Å^2^)	44.69	39.21	42.61	44.22
Number of TLS groups	9	48	10	44

For all three structures, the Rieske [2Fe-2S] cluster is well defined and coordinated by two histidine and two cysteine residues (Fig. 1*D*, Fig. S2*A*). This Rieske center is structurally conserved in all Rieske oxygenases, such as stachydrine demethylase Stc2 (28), naphthalene 1,2-dioxygenase NdoB (26), and dicamba monooxygenase DdmC (30) (Fig. S2*B*). The active sites containing mononuclear Fe centers are, however, considerably varied in enzymes of the Rieske oxygenase family (Fig. S2*B*), reflecting the diverse catalytic reactions catalyzed by these enzymes. The mononuclear Fe exists in an octahedral geometry and is coordinated by two histidine ligands (His213, His208) and a bidentate aspartate residue (Asp 323) in a well-documented His-His-Asp catalytic triad (31), coordinating one face of the Fe center (Fig. 1*E*). A pair of reduced Cys (Cys206, Cys209) is found near the mononuclear Fe in CntA, whereas in stachydrine demethylase Stc2 the corresponding Cys formed a disulfide bridge (28). A water molecule and a thiocyanate [SCN]^-^ anion occupy the two coordination sites, which are 2.2 and 2.3 Å away from the mononuclear Fe center in CntA, respectively (Fig. 1*E*, Fig. S2*C*). Both the water molecule and [SCN]^-^ are positioned 3.7 Å below the Cα adjacent to the carnitine ammonium group, which is expected to be the site of hydroxylation catalyzed by this enzyme (Fig. 1*E*). SCN- bound at the O_2_ site likely resulted in the stabilization of CntA cocrystalized with the substrates. The catalytic mononuclear Fe in purified CntA is in a ferrous (Fe^2+^) state, showing no EPR signals associated with either high-spin (*S* = 5/2) or low-spin (*S* = ½) ferric center (Fe^3+^) despite purifying the protein under aerobic conditions. This is confirmed by the addition of nitric oxide (NO) to CntA, showing an EPR signal from a *S* = ½ species of the Fe^2+^-NO adduct (Fig. 1*F*). This is contrast to the other nonheme oxygenase enzymes, where NO binding to the mononuclear iron center often led to the formation of high-spin, *S* = 3/2 signal (32). This plausibly suggests that the mononuclear iron ion exists as an *S* = 0 in its ferrous state. The observed EPR signal for the Fe^2+^-NO adduct is similar to the previously reported heme-/nonheme-NO adducts (33, 34).

### Structural basis of long-distance electron transfer and EPR spectroscopy

**Figure 2:**
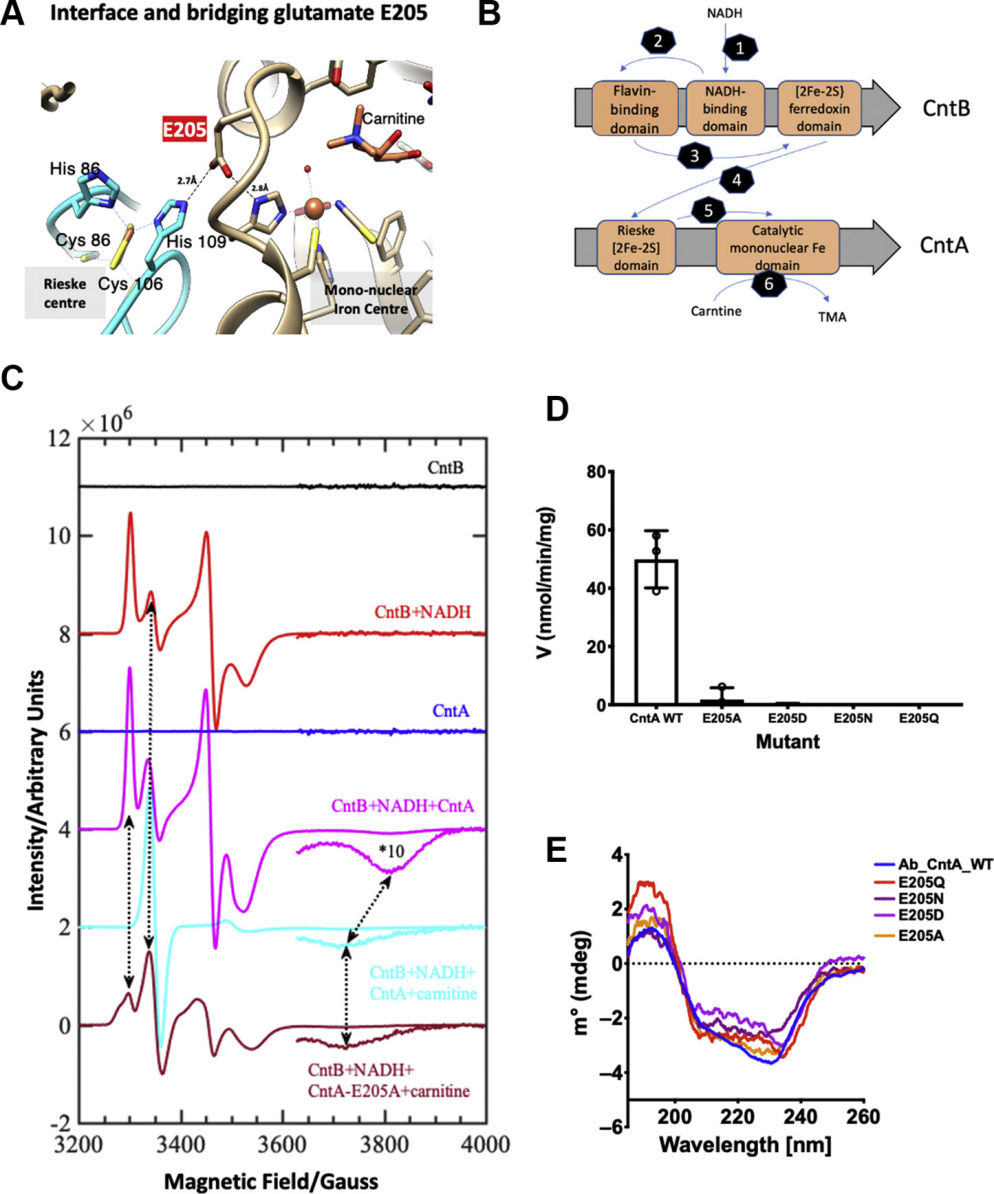
**Structural basis of intersubunit electron transfer and investigations by EPR.***A*, electron transfer in CntA between the Rieske center and the mononuclear Fe center across the interface of two neighboring CntA subunits (colored in *cyan* and *brown*, respectively) involved a key bridging glutamate reside (E205). Distances shown in angstrom. *B*, proposed electron transfer pathways from the reductant NADH to the catalytic mononuclear Fe center in CntA *via* the reductase CntB. *C*, EPR spectra of a series of combinations of WT CntAB proteins, E205A mutant, NADH, and L-carnitine to track the propagation of EPR signal and thus the inter-/intrasubunit electron transfer. NADH reduction in CntB (*black trace* to *red trace*; steps 2 and 3 in *B*); the EPR active [2Fe-2S]^+1^ species in the CntA Rieske center with and without carnitine present (*red trace* to *magenta trace* and *cyan trace*; steps 4–6 in *B*). The E205A mutant (*wine red trace*) demonstrates an EPR signal different from that of the wildtype CntA (*cyan trace*), indicating a disruption to the electron transfer pathway; EPR conditions, as described for Figure 1*F*. *D*, Activity assays of CntA E205 mutants. *E*, circular dichroism measurement comparisons between CntA wildtype and E205 mutant enzymes showing no difference in secondary structure.

Structural determination of CntA revealed that the two functional domains in CntA (the Rieske domain and the catalytic mononuclear Fe domain) are located 44 Å apart in the same subunit. As such, electron transfer to the catalytic mononuclear Fe center is likely to occur only at the interface of two adjacent CntA subunits (12.2 Å apart) and facilitated by the so-called bridging glutamate (E205, Fig. 2*A*) (13). Electron transfer from NADH to the carnitine oxygenase CntA is mediated through a flavin mononucleotide (FMN)-containing reductase CntB, which was essential for the enzyme activity (13). To monitor electron transfer pathway (Fig. 2*B*) from the reductant NADH *via* the flavin-containing reductase CntB to the catalytic domain in the oxygenase CntA, continuous wave (cw)-EPR was used.

Purified CntB is EPR-silent (Fig. 2*C*, black trace); however, the reduced ferredoxin center in CntB is readily detectable once NADH is added (red trace). The EPR spectrum of reduced CntB has a characteristic reduced ferredoxin [2Fe-2S]^+1^ signal [*g* tensor 2.031, 1.937, 1.899] and a radical signal resulting from flavin semiquinone g_ave_ = 2.0015. Similar flavin semiquinone radicals have been observed previously in other Rieske oxygenase reductase as well as monoamine oxidase (35, 36). Reduced CntB spectrum was simulated by a combination of two different *S* = ½ spin states (97% for the reduced ferredoxin and 3% for a flavin semiquinone radical) (Table 2), confirming electron transfer from the reductant NADH to the ferredoxin domain in CntB (steps 1–3 in Fig. 2*B*). The purified CntA (blue trace) showed a very weak anisotropic EPR signal with g_ave_ < 2.0 (*g* tensor 2.011, 1.916, 1.757), indicating that the Rieske center in CntA is predominantly in the oxidized, diferric [2Fe-2S]^2+^ state (Table 2) (37, 38). Addition of an oxidizing agent (H_2_O_2_) to CntA has minimal effect on the overall spectrum (Fig. S3*A*); however, the Rieske center in CntA can be readily reduced by dithionite (Fig. S3*B*). CntA also appears capable of oxidizing the substrate (carnitine) using H_2_O_2_ in the absence of CntB/NADH (Fig. S3*A*), consistent with the peroxide shunt-mechanism known in other Rieske oxygenases (39, 40). In order to monitor electron flow from CntB to CntA, the spectrum of CntA + NADH + CntB was recorded (magenta trace). This complex spectrum can be simulated by the combination of three different *S* = ½ spin states (Table 2, Fig. S4). Thus, the addition of CntB + NADH to CntA allowed reduction of the Rieske center in CntA, confirming cross-subunit electron transfer from CntB to CntA (step 4 in Fig. 2*B*).

**Table 2:** Spin-Hamiltonian parameters used to model the EPR spectra shown in Fig. 2*C*

Samples	EPR active signal	Species	%	*g*_1_	*g*_2_	*g*_3_	*g*_ave_
As-isolated CntA				2.0105	1.9157	1.7574	1.8945
As-isolated CntB	No EPR active signal detected						
CntB+NADH	[2Fe-2S]^+1^	specA (reduced ferredoxin domain)	93	2.0314	1.9372	1.8988	1.9558
	Flavin radical	specB (Flavin radical)	7	2.0015	2.0015	2.0015	2.0015
CntA+CntB+NADH	[2Fe-2S]^+1^ in CntB	specA (reduced ferredoxin domain)	61	2.0313	1.9376	1.8979	1.9556
	Flavin radical	specB	2	2.0010	2.0034	2.0039	2.0028
	[2Fe-2S]^+1^ in CntA	specC (reduced Rieske center)	37	2.0079	1.9153	1.7542	1.8924
CntA+CntB+NADH+Carnitine	an organic radical	specB	34	2.0000	2.0035	2.0037	2.0024
	[2Fe-2S]^+1^ in CntA^a^	specC	66	2.0071	1.9077	1.7986	1.9045
E205A+CntB+NADH+Carnitine	[2Fe-2S]^+1^ in CntB	specA1	40	2.0338	1.9397	1.8868	1.9534
	[2Fe-2S]^+1^ in CntB	specA2	20	2.0449	1.9493	1.9037	1.9660
	Flavin radical^b^	specB	8	2.0012	2.0040	2.0040	2.0031
	[2Fe-2S]^+1^ in CntA^a^	specC	32	2.0070	1.9077	1.7896	1.9014

a This EPR active signal may also contain overlapping EPR signals derived from the catalytic mononuclear iron center; plausible EPR active intermediates are ferric-(hydro)peroxy or a high-valent iron(V)-oxo species.

b This may represent an overlay of both a flavin and an unidentified organic radical.

We next sought to confirm the electron flow from the reduced Rieske cluster to the catalytic mononuclear Fe center in CntA and investigate the critical role of the bridging E205 in electron transfer as suggested by the CntA structure (step 5 in Fig. 2*B*). The E205 residue bridges the Rieske cluster on one subunit and the mononuclear Fe center in the adjacent subunit (Fig. 2*A*) (13, 41). None of the E205 mutants that we generated in this study were active (Fig. 2*D*), with no major perturbations to the overall secondary structure as observed by circular dichroism (Fig. 2*E*). As it is known that substrate binding can be required for the activation of the catalytic mononuclear Fe center to initiate catalysis (42, 43), the substrate (carnitine) was added to the reaction and the EPR spectrum was monitored (Fig. 2*C*, cyan trace). The spectrum shows no EPR signals at low magnetic fields, ruling out the presence of high-spin (*S* = 5/2 or 3/2) ferric species in the sample. However, strong isotropic (3350 G) and highly anisotropic EPR signals are observed between the magnetic fields of 3200 to 3900 G (Fig. 2*C*). The observed *g*_ave_ < 2.0 (Table 2) strongly indicates that these EPR transitions likely arise predominantly from one-electron reduced [2Fe-2S]^+1^ Rieske cluster in CntA and the isotropic signal at 3350 G likely originates from a new organic radical species, which is formed during CntA catalysis in the presence of the substrate. The EPR simulation from these two *S* = ½ species (reduced Rieske cluster and a new organic radical species) reproduced the observed spectra (Table 2, Fig. S4). The complete loss of reduced ferredoxin EPR signal from CntB, together with the formation of a novel organic radical species (isotropic signal seen at 3350 G) in CntA + CntB + NADH + carnitine suggests an efficient electron transfer to the catalytic mononuclear Fe in the wildtype CntA (step 5 in Fig. 2*B*).

To further support this proposed electron transfer pathway, we compared EPR spectra of the E205A mutant with the wildtype CntA. Indeed, the EPR spectra are significantly different between E205A (Fig. 2*C*, wine red trace) and the wildtype CntA (cyan trace). The spectrum of the E205A mutant shows complex EPR signals with four different *S* = ½, EPR active species (Table 2). In the spectrum with the E205A mutant, a significant amount (60%) of the EPR signal of the reduced [2Fe-2S]^1+^ ferredoxin in CntB was observed, whereas this was not found in the spectrum of the wildtype CntA, suggesting that electron flow is significantly impacted in this mutant. Together, the EPR observations provide strong evidence of each step involved in the electron flow as proposed in Figure 2*B* and confirm the role of the bridging glutamate (E205) in cross-subunit electron transfer as suggested by the CntA structure.

### Structural basis of substrate binding and inhibition

**Figure 3:**
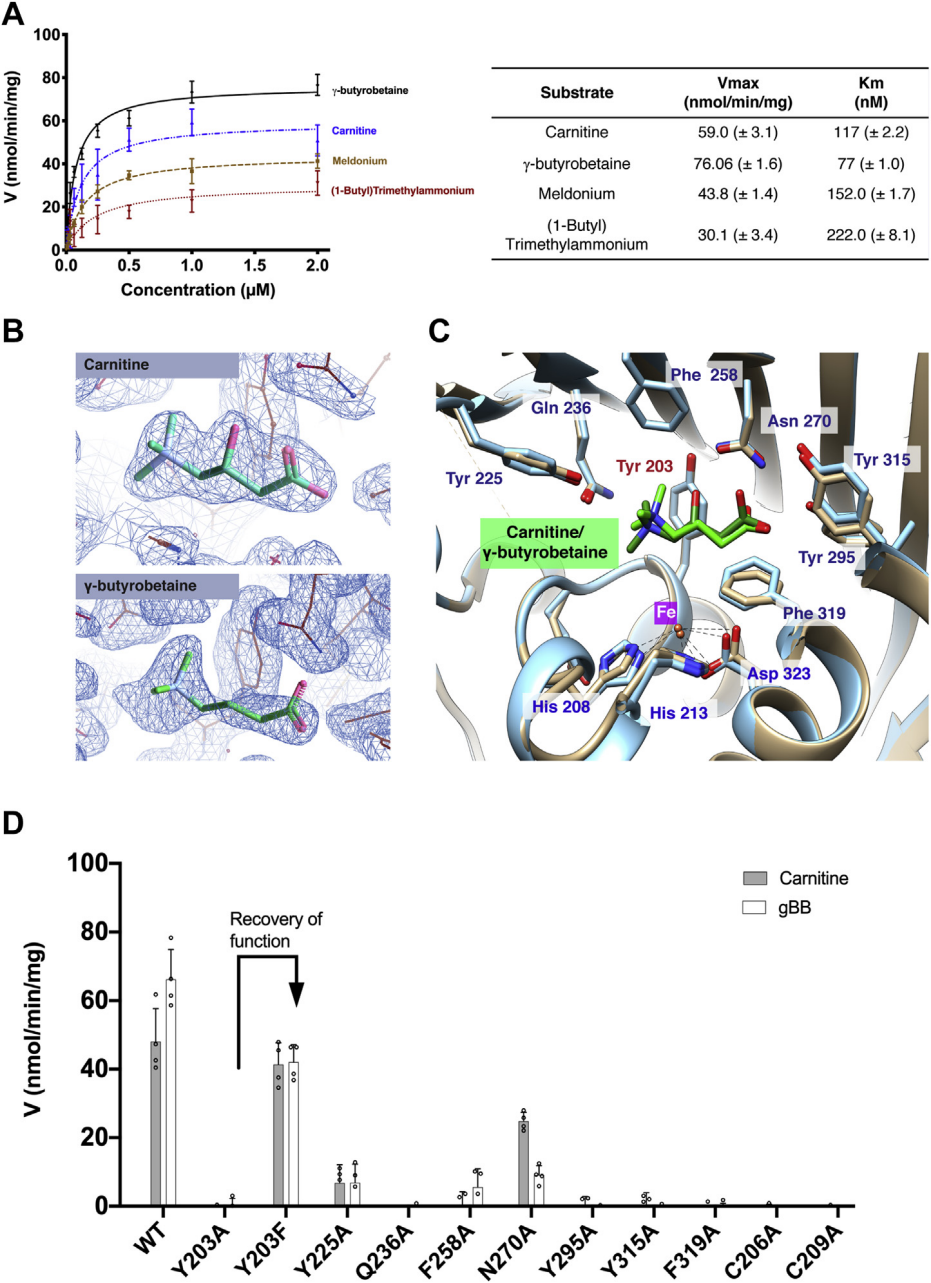
**CntA substrate binding pocket.***A*, Michaelis–Menten kinetics of CntA (n = 4). *B*, 2*mF*_o_−*DF*_c_ map at 1.5σ maps (*blue*) of carnitine displayed at 3.0σ and γ-butyrobetaine (gBB) displayed at 2.5σ. *C*, carnitine and γ-butyrobetaine interacting residues in the active site among which Y203 interacts with the substrate of CntA through the π–cation interaction. *D*, enzyme activity of site-directed mutants of key residues involved in substrate coordination in CntA from N = 3 independent replicates. The Y203F mutant in CntA regains activity alluding to the crucial role of the aromatic system for a π–cation interaction.

CntA can catalyze the oxidation of carnitine and several substrate analogs (Fig. 3*A*). The high-resolution CntA structures cocrystalized with the ligands (carnitine, 2.0 Å; gBB, 1.6 Å) allowed us to define the substrate-binding pockets. The ligands were built into the clear density, which was much better defined for the carnitine structure (Fig. 3*B*). The substrate-binding site is formed by a series of β sheets and two α helices coordinating the substrate through hydrophobic/steric interactions involving residues Phe258, Tyr315, and Phe319 and polar interactions with residues Tyr225, Asn270, and Tyr295. (Fig. 3*C*, Fig. S5). The Phe319 residue is positioned at the kink of an observed π-helix feature for residues 306 to 337 (44). Importantly, we also observe that Tyr203 forms a π–cation interaction with the positively charged ammonium group in the substrates (Fig. 3*C*). The importance of the binding site residues was then tested by mutating them to alanine. Indeed, all mutants decreased or abolished activity (Fig. 3*D*). Interestingly, the Y203F mutant was active (Fig. 3*D*), demonstrating a recovery of function and the pivotal role of the π–cation interaction with the ammonium group.

**Figure 4:**
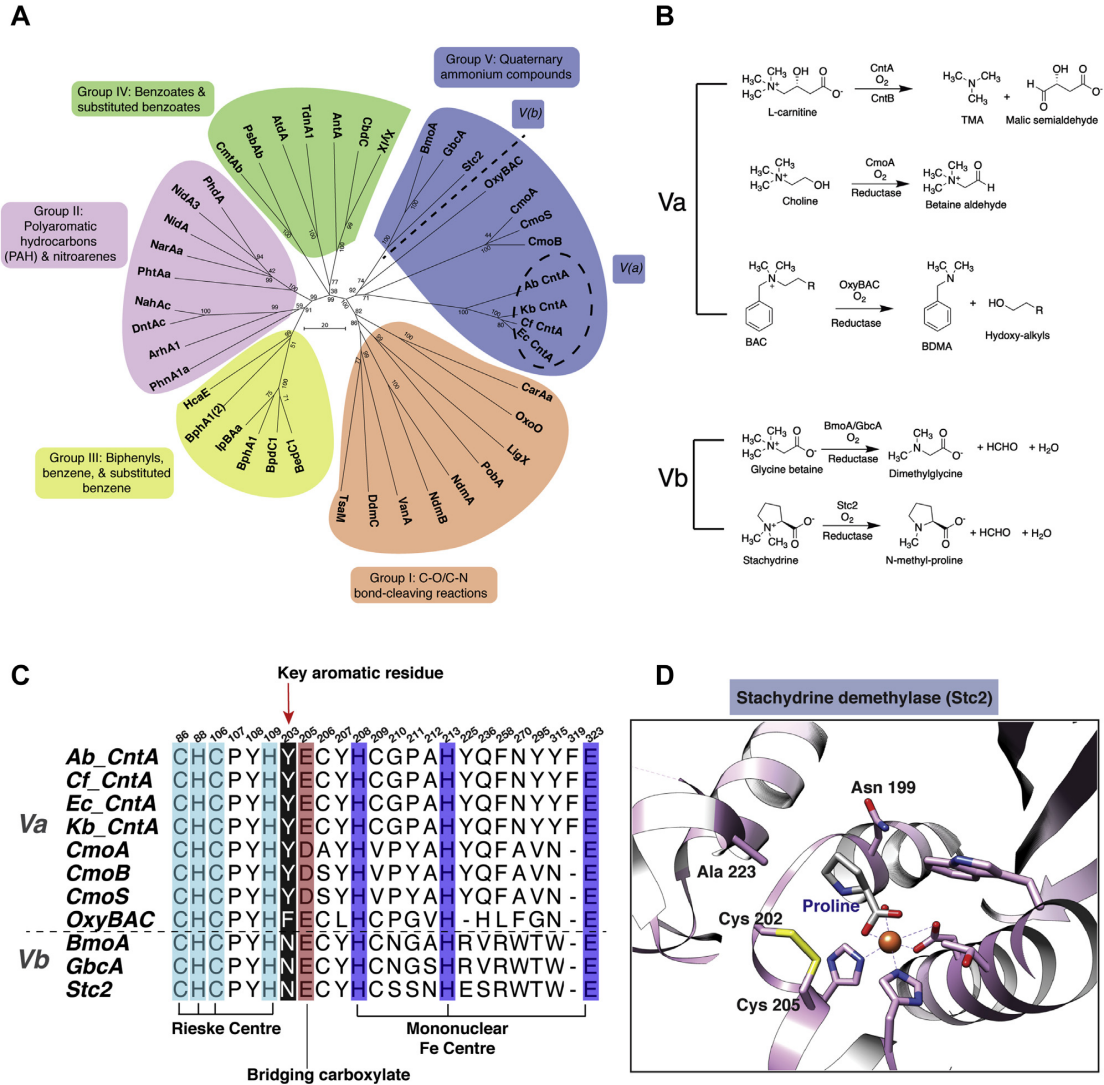
**CntA represents a novel group of Rieske oxygenases using quaternary amine substrates (group V).***A*, phylogeny of Rieske oxygenases showing the five distinct groups. The evolutionary history of Rieske oxygenases was inferred using the Neighbor-Joining method in MEGA7 (68). The *dashed circle* grouping shows the clustering of CntA including *Acinetobacter baumannii* (Ab), *Escherichia coli* SE11 (Ec), *Klebsiella pneumonia* (Kb), and *Citrobacter freundii* (Cf) (see supplementary information, Appendix 1 for a list of gene accession ID’s). Group V Rieske oxygenases oxidize quaternary amine substrates, including carnitine (CntA), choline (CmoA/B/C), benzalkonium (OxyBAC), glycine betaine (BmoA, GbcA), and stachydrine (Stc2). *B*, chemical reactions catalyzed by Rieske enzymes from the Group V Rieske oxygenases involved in quaternary amine oxidations. Group Va includes carnitine oxygenase (CntA) choline monooxygenase (CmoA, CmoS, and CmoB), and benzalkonium oxygenase (OxyBAC). Group Vb carries out oxidative demethylation reactions. *C*, sequence alignment of the substrate-binding pocket residues of group V Rieske oxygenase involved in quaternary amine oxidation. The aromatic residues for substrate coordination through a π–cation in group Va and the corresponding position in group Vb are highlighted by a *red arrow*. The Rieske center is coded in *blue*, the catalytic triad of the mononuclear Fe center is shown in *purple*, and the bridging carboxylate is highlighted in *pink*. *D*, the substrate-binding site of the group Vb quaternary amine degrading enzyme Stc2. The substrate mimics proline in Stc2 coordinates with the catalytic mononuclear Fe center *via* a carboxylic acid group.

The presence of an aromatic residue in this position (Y203 or F203) appears to be restricted to CntA and several closely related enzymes. Indeed, a comprehensive phylogenetic analysis of Rieske oxygenases, including several other recently defined Rieske enzymes, involved in the oxidation of quaternary ammonium substrates clearly defined a group that delineates quaternary ammonium Rieske oxygenases (group V) from the well-studied ring-hydroxylating Rieske oxygenases (Fig. 4*A*) (27, 29). Groups I to IV Rieske oxygenases primarily catalyze the dihydroxylation of aromatic and polyaromatic compounds that are important in bioremediation of environmental pollutants, whereas group V Rieske oxygenases include enzymes that principally oxidize quaternary ammonium substrates (Fig. 4*B*). Interestingly, based on the presence or absence of the signature aromatic residue at position 203 (Y203 or F203), group V Rieske oxygenases can be further separated into two clades Va and Vb where in clade Vb the Tyr (or Phe in OxyBAC) is replaced by an Asn (Fig. 4*C*). Substrates for the former clade include carnitine (CntA (13)), choline (CmoA, CmoS, CmoB (45, 46, 47)), and benzalkonium (OxyBAC (29)), whereas the latter clade Vb enzymes undertake oxidative demethylation from the ammonium group for glycine betaine (GbcA, BmoA (27)) and proline betaine (stachydrine, Stc2 (28)).

Comparison of the ligand-bound structures of CntA (group Va) and Stc2 (group Vb) uncovers striking differences in the way quaternary amine substrates are oriented (28). In Stc2 the substrate analog proline is ligated with the catalytic Fe through the carboxylate group (Fig. 4*D*), whereas in CntA, although a carboxylate group is present, the substrates were oriented *via* a π–cation interaction with the trimethylammonium moiety and the carboxylate group is not ligated with the mononuclear Fe (Fig. 3*C*).

**Figure 5:**
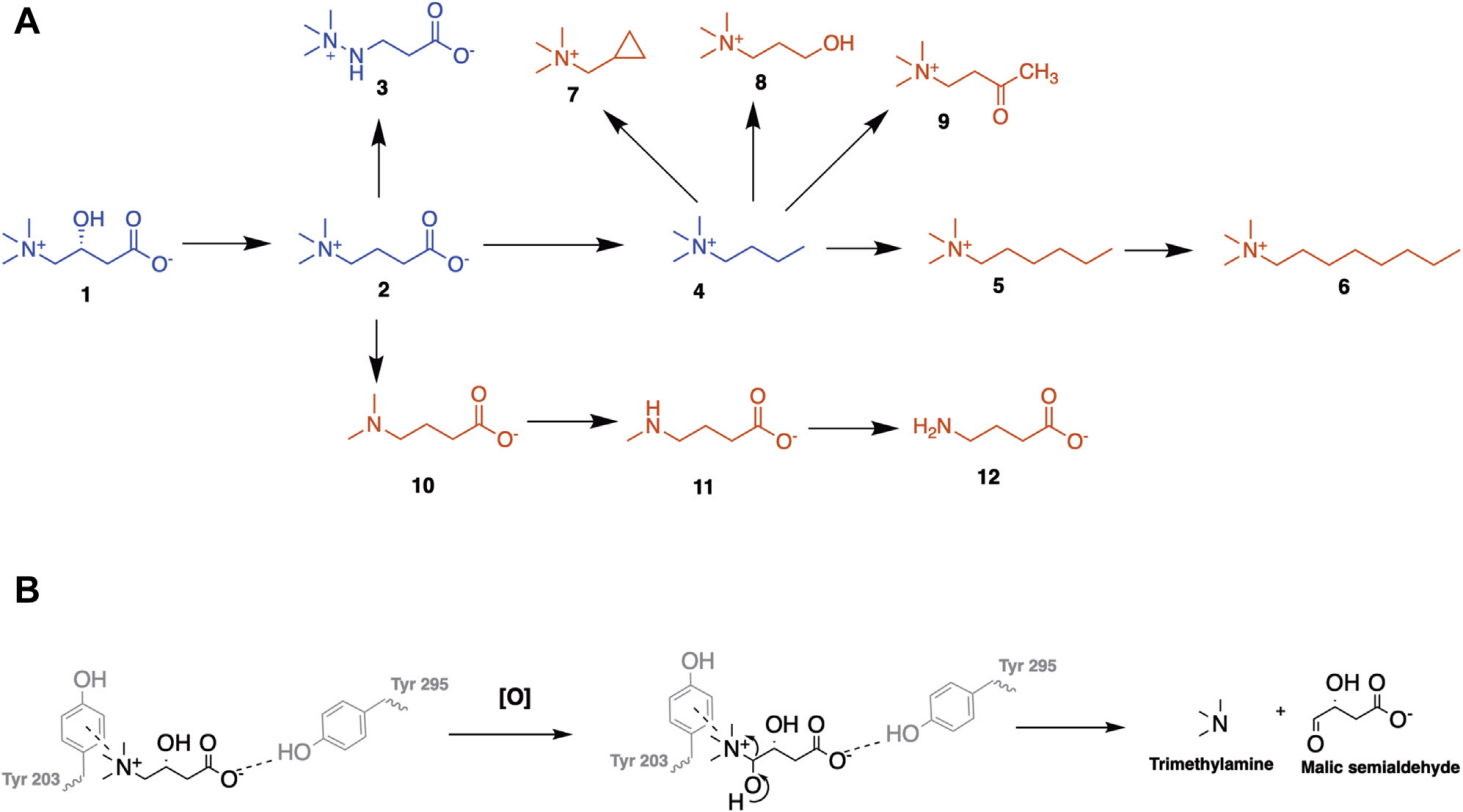
**Structure–activity relationship of CntA substrate profiling and coordination during catalysis.***A*, a structure–activity relationship scaffold hopping map of substrate analogs tested with a single change between structures and the progression shown with *arrows*. Structures in *blue* represent active substrates, whereas those in *red* are inactive. (1) Carnitine, (2) g-BB, (3) Meldonium, (4) (1-Butyl)Trimethylammonium, (5) (1-Hexyl)Trimethylammonium, (6) (1-Octyl)Trimethylammonium, (7) (Cyclopropylmethyl)-Trimethylammonium, (8) (3-Hydroxypropyl)Trimethylammonium, (9) (3-Oxobutyl)Trimethylammonium, (10) 4-(Dimethylamino)butyric acid, (11) 4-(Methylamino)butyric acid and (12) gamma-Aminobutyric acid. *B*, the coordination of quaternary amine substrates in the CntA-binding site during catalysis showing the role played by Tyr203 and Tyr295 in coordinating the substrates.

In order to understand the substrate specificity for the enzyme, we investigated the structure–activity relationship of 22 substrate analogs with differences on the aliphatic chain, the carboxyl group, or the amine terminal groups (Fig. 5*A*, Fig. S6). In addition to oxidizing carnitine/γ-butyrobetaine (compound 1 and 2), CntA can also oxidize meldonium (compound 3) and 1-butyl-trimethylammonium (compound 4) albeit with a lower affinity (Fig. 3*A*, Fig. 5*A*). The lack of a carboxylic acid in compound 4 reduces the affinity for the substrate, and substrates with longer alkyl chains or other functional groups showed no activity (compounds 5–9). A further series of compounds related to γ-butyrobetaine but not quaternary ammonium salts (compounds 10–12) did not serve as substrates for CntA. Other quaternary amine compounds tested were not accepted as substrates (Fig. S6). Together, structural, mutagenesis, and structure–activity relationship data therefore suggest that the substrates for CntA are primarily coordinated through a π–cation interaction between Y203 and the trimethylammonium group, which positions the substrate in place above the mononuclear Fe site for oxidative cleavage to occur (Fig. 5*B*). Although there is some tolerance for the lack of a carboxylic acid, increases in the length of the molecule and increased steric bulk are not favored for enzyme activity as they likely disturb the polar interaction with Tyr295.

### A competitive inhibitor binds to the same substrate recognition pocket through π–π stacking interactions

**Figure 6:**
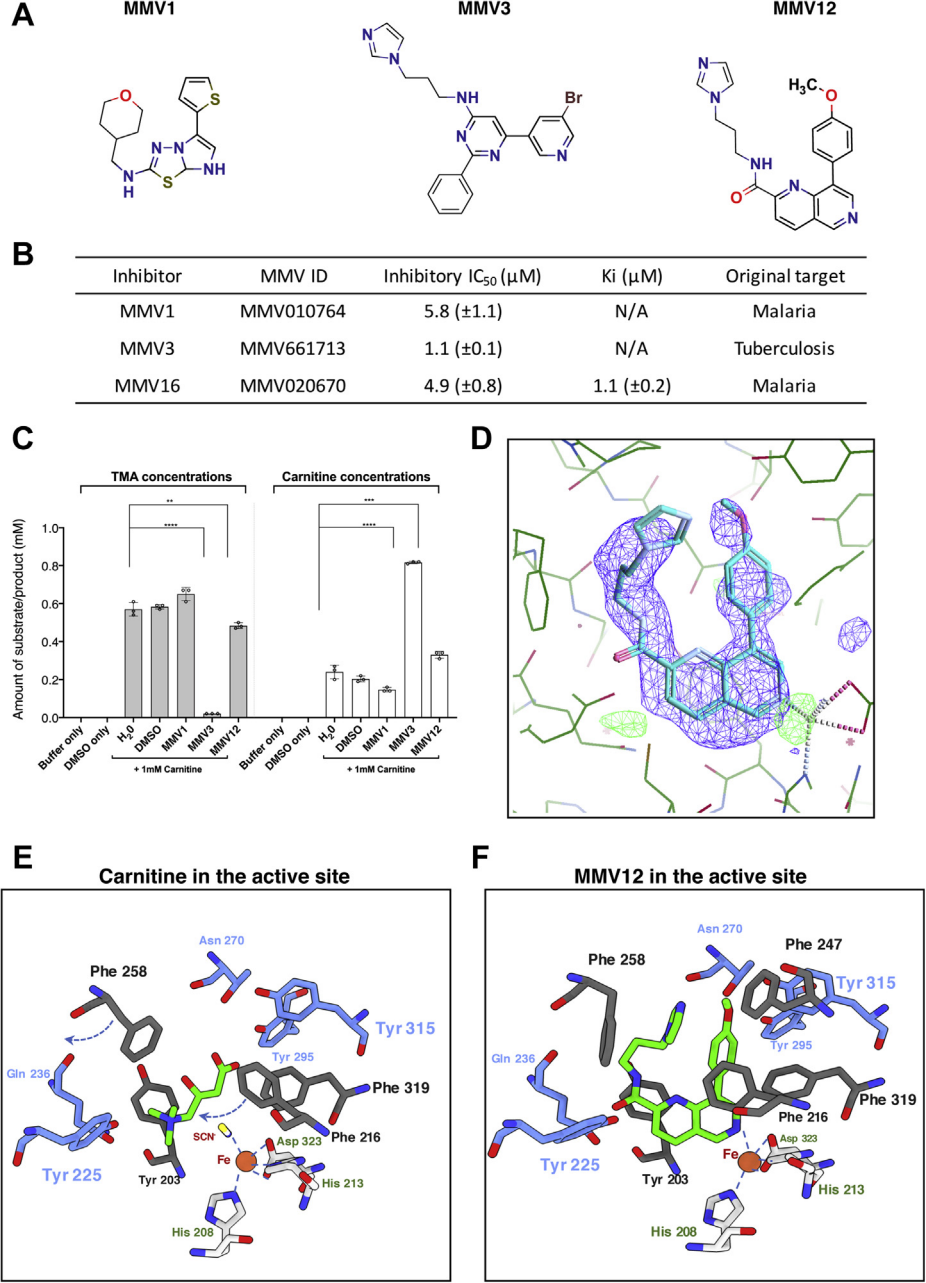
**Structural basis of CntA inhibition.***A*, chemical structures of the three CntA inhibitors: (MMV1), MMV010764 (N-[(oxan-4-yl)methyl]-5-(thiophen-2-yl)imidazo[2,1-b][1,3,4]thiadiazol-2-amine); (MMV3), MMV661713(6-(5-bromopyridin-3-yl)-N-[3-(1H-imidazol-1-yl)propyl]-2-phenylpyrimidin-4-amine); (MMV12), MMV020670 (N-[3-(1H-imidazol-1-yl)propyl]-8-methyl-1,6-naphthyridine-2-carboxamide). *B*, summary of the inhibitor assays with inhibitory IC_50_ data values shown for n = 3 replicates. *C*, evaluation of CntA protein inhibitors (MMV1, MMV3, MMV12) using *A. baumannii* bacterial cultures. Cells treated with inhibitors MMV3 (*p* < 0.0001) and MMV12 (*p* < 0.001) caused significant reduction in trimethylamine (TMA) formation from carnitine, whereas MMV1 did not reduce TMA production. Carnitine was added to a final concentration of 1 mM prior to the incubations with/without additional inhibitors. Control runs of buffer only and DMSO only did not have carnitine in the treatment, and no TMA was observed. N = 3 for all conditions. Statistics was carried out using one-way ANOVA. ∗∗*p* < 0.01; ∗∗∗*p* < 0.001; ∗∗∗∗*p* < 0.0001. *D*, A Polder OMIT map (*blue*) of MMV12 (MMV020670) displayed at 3.0σ in the CntA active site. *E*–*F*, comparison of selected residues in the CntA active site interacting with the carnitine substrate and MMV12 (MMV020670) inhibitor. Polar interacting residues (*magenta*) remain unchanged, whereas for the hydrophobic π–π interacting residues (*dark gray*), positional changes for Phe258 and Phe216 as indicated by *dashed arrows* in *E*.

Encouraged by previous success in the identification of substrate mimics as potent inhibitors for the choline-TMA lyase CutC (17, 18, 19, 20), we also screened these carnitine analogs (Fig. 5*A*, Fig. S6) as potential inhibitors of CntA in competition assays. However, none of these substrate analogs inhibited CntA activity. We thus used a random approach by screening drug libraries and successfully obtained three inhibitor compounds MMV1, MMV3, and MMV12 (Fig. 6*A*). Their inhibitory IC_50_ values are in the range of 1.1 to 5.8 μM (Fig. 6*B*). From competition assay data (Fig. S7) we were able to derive a K_i_ (1.09 μM) for MMV12 (ID, MMV020670), indicating that it is likely a competitive inhibitor.

We successfully solved the structure of MMV12 with CntA (PDB, 6ZGP), and indeed, the inhibitor occupied the substrate-binding pocket (Fig. 6*D*). Interestingly, Tyr203 and Tyr295, both of which are important for the coordination of carnitine in CntA (Fig. 5*B*), also play a key role in binding to the inhibitor (Fig. S8). Tyr203 forms a π–π stacking interaction with the 1,6-naphthyridine core ring, and Tyr295 forms polar interaction with the oxygen on the methoxy group in the inhibitor. Interestingly, the inhibitor is also coordinated to the mononuclear Fe *via* a nitrogen on the 1,6-naphthyridine ring at distance of 2.65 Å and to an oxygen of the carboxylic acid moiety of Asp323 at 2.7 Å (Fig. S8). Other π–π interactions are also observed between Phe319 and the phenyl methoxy group and between Phe258/Phe247 and the imidazole group. When the binding pockets of carnitine and MMV12 are compared (Fig. 6, *E*–*F*), Phe258 moves to accommodate the imidazole group of the inhibitor, whereas the loop containing Phe216 is drawn in closer to the active site to form the π–π interaction with the inhibitor.

The two other inhibitors MMV1 and MMV3 share similar structural features to MMV12. MMV1 has a core bicyclic fused aromatic scaffold with two branching cyclic groups. MMV3 has an imidazole group with an aliphatic amine linker group. The remaining pair of inhibitors show more complex patterns of inhibition for CntA. Using the structure of CntA cocrystalized with MMV12, we applied *in silico* docking of MMV1 and MMV3 to observe if these molecules could adopt comparable poses (Fig. S9). The results showed that both compounds show logical poses in the binding site with docking scores of −10.576 and −11.169 (kcal/mol), respectively. These are comparable with a score of −11.7 (kcal/mol) when MMV12 is docked into the empty pocket in CntA. Both docked compounds demonstrate likely coordination to the Fe center *via* a nitrogen atom and similar π–π stacking interactions.

In order to assess the activity of these inhibitors, we monitored the impact of these inhibitors on the production of TMA in *A. baumannii* cells pregrown on carnitine. Indeed, we observed a decrease in TMA production in the presence of MMV3 and MMV12 (Fig. 6*C*), whereas MMV1 did not inhibit TMA formation in the bacterial culture. MMV3 has almost completely inhibited TMA formation, whereas MMV12 caused ∼20% reduction in TMA production. It is worth mentioning that MMV3 was originally developed for treating tuberculosis and the differences in inhibitor activity toward *A. baumannii* cells may reflect their ability to penetrate bacterial cell membranes.

## Discussion

A major breakthrough in gut microbiome research over the past decade is the realization that gut microbiota play an integral role in human health and diseases (48). This is exemplified in the study of TMA formation from gut microbiota and its implication in the development of atherosclerosis and cardiovascular diseases (9, 10, 11). Recent progress using experimental animals and human clinical studies have shown that TMA precursors such as choline, carnitine, and γ-butyrobetaine promote atherosclerosis *via* the TMAO pathway through the interplay between gut microbiota and host metabolism (49). The identification of the key microbial enzymes, including the choline-TMA lyase (12) and CntA (13), provides promising new drug targets for attenuating TMA release from gut microbial metabolism of TMA precursors. Although much has been learnt from the structures of choline-TMA lyase and subsequent development of selective inhibitors for choline-to-TMA transformation (17, 18, 19, 20), little progress has been made on CntA owing to the lack of CntA structures. Here, in this study, we provide the structural basis of substrate coordination and mode of inhibition by the newly discovered inhibitors. The structure and EPR analysis also allow us to better understand the structural basis of long-range electron transfer across the interface of CntA subunits.

Rieske oxygenases are challenging proteins to study, and this was no different in the case of CntA. Different expression strategies or the choice of buffers and additives may have contributed to the apparent differences in enzyme activities among studies of CntA protein (13, 50). Owing to sensitivity to oxygen, considerable effort was made in our work in order to obtain CntA crystals. It is worth noting that the presence of SCN in the crystallization media was essential to obtain CntA crystals. Given that we observe the SCN coordinated to the Fe center in CntA-carnitine and CntA-gBB complex, we investigated the effect of additional SCN on the activity of CntA. Indeed, SCN inhibited CntA activity with the half maximal inhibitory concentration (IC_50_) of 35 mM (Fig. S10*A*) and EPR analysis showed that the addition of SCN may have affected inter-/intrasubunit electron transfer (Fig. S10*B*). It is therefore likely that SCN affects CntA functionality by coordinating to the Fe center and preventing oxygen binding, which in turn helps to stabilize the protein for crystallization.

Our study shows that CntA forms a head-to-tail homotrimer and the substrates appear to be poised in place next to the mononuclear Fe center ready for cleavage. Despite both substrates (*i.e.*, carnitine and γ-butyrobetaine) possessing carboxylate moieties, these are not coordinated to the mononuclear Fe center as might be expected and that has been observed for group Vb Rieske oxygenase such as proline-bound stachydrine demethylase Stc2 (28). The Y203 residue is strictly conserved in all CntA sequences, which is key for a π–cation interaction to coordinate the ammonium moiety of the substrate and for a π–π stacking interaction to the inhibitor (Fig. 6). This corresponding aromatic residue is also present in other group Va Rieske oxygenases, such as choline monooxygenase (tyrosine) and OxyBAC (phenylalanine) (Fig. 4), suggesting that such a π–cation interaction is commonly used for orientating choline and benzalkonium during catalysis. Conversely, when compared with the stachydrine and glycine betaine degrading enzymes, Stc2 and BmoA/GbcA, respectively, there is not an aromatic residue present in the same position. The structure for Stc2 showed that stachydrine coordinates to the mononuclear Fe through the carboxylate group, and the suggested mechanism requires this binding mode in order to cleave the methyl substituent (28). This insight suggests a differentiation among the group V members and thus a distinctive way to classify quaternary ammonium catalysis by such Rieske oxygenases.

Among the substrate analogs tested for CntA, activity was seen only in compounds containing a quaternary ammonium group, in agreement with the pivotal role of the π–cation interaction for substrate coordination. Given the link between TMA production and cardiovascular diseases, targeting CntA with small molecule inhibitors could have a therapeutic potential. Inhibitors for CntA may also be useful to treat individuals with a socially challenging condition trimethylaminuria or commonly referred to as the “fish odor syndrome” (51). An elegant example of such rational designed inhibitors starting from the endogenous substrate has been reported for the choline-TMA lyase, CutC with DMB (3,3-dimethyl-1-butanol), DMBA (3,3-dimethylbutyric aldehyde), FMC (N-(fluoromethyl)-2-hydroxy-N,N-dimethylethan-1-aminium), and (3S)-1-methyl-3,6-dihydro-2H-pyridin-3-ol (17, 18, 19, 20). FMC is reported as the most potent inhibitor with an EC_50_ of 56 nM by forming an irreversible covalent bond in the CutC catalytic site (19). Encouragingly and particularly relevant to CntA is the development of inhibitors based on the structure of γ-butyrobetaine against the carnitine-producing γ-butyrobetaine hydroxylase from human (52). The inhibitors were derivatives of the trimethylammonium, aliphatic chain, and carboxylate region, with some demonstrating a similar binding mode to the native substrate but with higher affinities. The structure of CntA, together with the information inferred from the substrate mimics and the competitive inhibitors that we reported here in this study, could be investigated in a rational drug design approach to formulate more potent CntA inhibitors for future exploitation. The data presented here suggest that CntA substrate analogs are less likely to inhibit its activity and rational drug design should perhaps take advantage of the presence of several aromatic residues in the binding pocket to utilize a π–π stacking interaction as we have demonstrated in the inhibitor compound (Fig. 6).

Taking advantage of the CntA structure we solved in this study, we employed EPR analysis to track the activation and progression of the electron transfer pathway involving the carnitine oxygenase complex. We propose that, during catalysis, CntA and its associated reductase, CntB, approach and electron transfer from the one-electron reduced ferredoxin [2Fe-2S]^+1^ in CntB to the oxidized Rieske [2Fe-2S]^2+^ effectively takes place. Efficient electron transfer from the reduced Rieske [2Fe-2S]^1+^ center to the catalytic mononuclear Fe center in CntA requires both substrate, carnitine, and the crucial bridging glutamate reside E205, mutation of which significantly alters electron flow (Fig. 2*C*), resulting in the loss of enzyme activity (Fig. 2*D*). This was also independently validated by a recent study showing that both E205D and E205Q mutants lost enzyme activity and the ability for electron transfer to the active center (13, 50). Binding of the substrate to the catalytic mononuclear Fe center caused a shift in *g*_*3*_ tensor from 3810 G to 3750 G (Fig. 2*C*). This suggests that substrate entrance might influence the redox potential of the mononuclear Fe center in CntA, possibly influencing the electron transfer from the reduced Rieske center to the mononuclear Fe center in CntA, although its impact on the one-electron reduced Rieske center cannot be completely ruled out. The terminal step of the electron transfer pathway is the formation of an organic radical species (Table 2), which is likely to be crucial for CntA catalysis. Its identity, however, has yet to be elucidated. Potential active intermediates in other well-studied Rieske oxygenases include Fe(III)-(hydro)peroxy followed by a rearrangement leading to a high-valent hydroxo-Fe(V)=O or Fe(IV)=O species (53, 54, 55). The active high-valent catalytic Fe in CntA catalysis certainly warrants further investigations. However, the methodology established here may also facilitate the discovery of novel CntA inhibitors targeting the electron transfer pathway.

To sum up, this work provides the structural basis of carnitine degradation by CntA. Structural determination of CntA and spectroscopic characterization using EPR provides structural and mechanistic insight into the role of bridging glutamate (E205) in intersubunit electron transfer. Cocrystallization of the substrates and the competitive inhibitor with CntA unveils the role of the π–π stacking interaction as the key mode of inhibition. The work presented here provides the structural basis to facilitate rational design and further optimization of specific inhibitors targeting carnitine-to-TMA transformation by gut microbiota.

## Experimental procedures

### Cloning of CntAB and site-directed mutagenesis of CntA

The plasmids for expressing CntA and CntB of *A. baumannii* were constructed previously (13). All *A. baumannii* CntA mutants were chemically synthesized by GenScript and cloned into the pET28a(+) expression vector using the NdeI and HindIII sites. All plasmids were transformed into BL21(DE3)pLysS cells for protein expression as described previously (13).

### Protein expression and purification

For each respective protein, recombinant *Escherichia coli* strains were streaked from glycerol stocks onto LB agar plates with kanamycin (50 μg/ml) and incubated overnight at 37 °C. Recombinant protein expression was induced with 0.2 mM IPTG into liquid culture in LB medium. After 18 h incubation the cells were collected by centrifugation at 6000 rpm at 4 °C for 10 min and the pellet was resuspended in 20 ml PBS + 0.3 M NaCl on ice, aliquoted and frozen at −80 °C. For purification, the standard buffer conditions were 20 mM Tris-HCl pH 7.6, 250 mM NaCl, and 0.5 mM tris(2-carboxyethyl)-phosphine (TCEP). To purify CntA and CntB proteins, cells were broken by sonication in the standard buffer and the cell-free extract was loaded on a Roche Complete resin for affinity purification. The resin was then washed with standard buffer + 5 mM imidazole, and the target protein was eluted with the standard buffer + 250 mM imidazole + 10% (v/v) glycerol. The elution was concentrated in a Vivaspin 20 10,000 kDa cutoff to 2.2 ml before loading onto a Superdex 200 16/600 column using an ÄKTA purifier and buffer exchanging into 10 mM Hepes, 250 mM NaCl, 0.5 mM TCEP, and 10% glycerol (v/v) buffer for storage or 10 mM Hepes pH 7.6, 10 mM NaCl, and 0.5 mM TCEP for crystal growth. For storage, the protein was concentrated to 2 mg/ml in a Vivaspin 6 10,000 kDa and aliquots were kept frozen at −20 °C; for crystallography the protein was concentrated to the desired amount (between 5 and 50 mg/ml) and used immediately in crystallization setups. Purified CntB was estimated to have ∼0.85 FMN per CntB using the method of Batie *et al.* (35) by measuring absorbance at 462 nm against FMN standards ranging from 0.3 to 321.5 μM.

### Protein crystallography

CntA crystals were seeded and obtained at 22 °C using the hanging drop method. All drops contained 1 μl of protein material and 1 μl crystallization condition. Initial crystallization conditions were obtained from sparse matrix screening using JCSG-plus (Molecular Dimensions) and optimized using the Additive Screen (Hampton Research). Seeding material was generated with protein at 10 to 20 mg/ml in drops containing 100 mM Hepes, 0.2 M NaSCN, 20% (w/v) PEG 3350, 10 mM NaCl, and 0.5 mM TCEP where large irregular crystals formed over 48 h at 22 °C. These crystals were scratched with acupuncture needles and then streaked through drops containing protein at 7.5 mg/ml 100 mM HEPES, 0.2 M NaSCN, 18% (w/v) PEG 3350, 10 mM NaCl, and 0.5 mM TCEP with substrates (carnitine and γ-butyrobetaine) also present at a final concentration of 1 mM; the needle was run through 2 consecutive drops with a third drop on the cover slip as a control. Single red hexagonal crystals developed between 24 and 36 h at 22 °C and were collected immediately thereafter (prolonged incubation of the crystals was found to be detrimental to their crystal quality). Crystals were cryoprotected in an equivalent solution to the mother liquor supplemented with 5% glycerol and flash cooled in liquid nitrogen. For CntA + MMV020670, MMV020670 was added to a final concentration of 250 μM in the crystallization condition buffer drops that were streaked and yielded the same red hexagonal crystals 36 to 48 h after setting up. The crystals were collected and stored as mentioned above.

The crystals were mounted robotically on the i24 beamline (apo, carnitine, and γ-butyrobetaine) and i04 beamline (CntA + MMV020670) at the Diamond Light Source (Harwell Science and Innovation Campus, Didcot, UK). In the apo and γ-butyrobetaine collections, we observed split spots and streaking of spots on some images. The apo, carnitine, and MMV020670 data were auto-processed with Dials (56) using the Xia2 pipeline at Diamond Light Source followed by scaling with Aimless (57). For the γ-butyrobetaine dataset we processed the data using the Dials User Interface followed by scaling with Aimless in the CCP4i2 suite of tools (58). An initial model was derived from PDB 3VCP. Molecular replacement was performed in PHASER. Autobuilding in phenix.autobuild was followed by iterative rounds of manual building in COOT (59) interspersed with refinement in PHENIX (60). Within Coot, monomers for L-carnitine (PDB code 152), γ-butyrobetaine (PDB code NM2), glycerol (GOL), thiocyanate (SCN), and Hepes (EPE) were added into clear density that was evident in both 2fo-fc and fo-fc maps consistent with the respective ligands. In order to assign the coordinates for MMV020670, Polder OMIT maps (61) were generated in PHENIX. Geometry restraint information as .cif file for MMV020670 was generated in eLBOW (62) within the PHENIX suite of programs and the MMV020670 inhibitor was added into the model guided by Polder OMIT maps. The structure model coordinates can be accessed from the PDB repository with the following accession codes: CntA Apo (6Y8J), CntA + carnitine (6Y9D), CntA + gBB (6Y8S), and CntA+MMV020670 (6ZGP).

### UV-visible enzyme assays

All activity assays were performed on a BMG FLOUstar Omega 96-well plate reader, set at wavelength 340 nm with a sample volume of 200 μl. The assay comprised combining mixture A 60 μg CntA and 60 μg CntB with mixture B 0.25 mM L-carnitine and 0.25 mM NADH in a 10 mM Hepes buffer, pH 7.6 with 250 mM NaCl and 0.5 mM TCEP. The enzyme does not accept NADPH as the electron donor, and the additional flavin (FAD or FMN) to the enzyme assay mixture had no major impact on enzyme activities (Fig. S1). The reactions were measured for 5 min recording the linear decrease of signal at 340 nm for NADH oxidation. The slope of the line was measured between 60 and 120 s to yield the rate.

### Michaelis–Menten kinetics

Michaelis–Menten kinetic parameters were derived using the UV-visible enzyme assays methodology above. Substrate concentrations were varied from a top concentration of 2 mM and decreasing in a twofold manner for 10 successive dilutions. Observed rates were obtained from four independent measurements and fitted to a nonlinear regression Michaelis–Menten model in Prism 8 for macOS v8.2.0.

### Electron paramagnetic resonance analysis

EPR measurements were carried out using a Bruker ELEXSYS-E500/E580 X-band EPR spectrometer operating in both cw and pulsed modes, equipped with an Oxford variable-temperature unit and ESR900 cryostat with Super High-Q resonator as reported previously (63, 64). All EPR samples were prepared in the quartz capillary tubes (outer diameter, 4.0 mm; inner diameter, 3.0 mm) and flash frozen immediately and stored in liquid N_2_ until analysis. The X-band EPR tubes were then transferred into the EPR probehead, which was precooled to 20 K. The low-temperature EPR spectra were measured at 20 K as a frozen solution. A microwave power of 30 dB (0.2 mW) and modulation of 5 G appear to be optimal for recording the EPR spectrum of the CntA/CntB (oxygenase/reductase) domains prepared under various experimental conditions. The concentration of the CntA/CntB domains was 200 μM in all the samples. All experiments were carried out in 10 mM Hepes, pH 7.6, 250 mM NaCl, 10% glycerol, and 0.5 mM TCEP, with additional NADH and or L-carnitine at a final concentration of 75 mM added accordingly. The analysis of the cw-EPR spectra and simulations were performed using EasySpin toolbox (5.2.18) for the MATLAB program package (65).

### Circular dichroism

The proteins were buffer exchanged on a PD-10 column into a 0.2 M sodium phosphate buffer with 0.01 M NaCl at pH 7.0 and prepared at 0.1 mg/ml final concentration. The samples were analyzed on a JASCO J-1500 at 20 °C using a 0.1-mm path length quartz cuvette. Data were collected between 260 and 180 nm with eight scans per sample collected.

### Inhibitor screening assay

Compounds in the Pathogen Box (https://www.mmv.org/mmv-open/pathogen-box/about-pathogen-box) were received as 10 mM stocks dissolved in dimethylsulfoxide (DMSO). The compounds were added such that the final concentration was 50 μM in the 200-μl reaction. As per the “UV-visible enzyme assays” methodology above, the protein in mixture A was incubated with inhibitor compounds for 20 min before commencing the assay. Compounds that demonstrated 25% or better inhibition relative to an untreated and equivalent DMSO control were further validated in two successive independent assays to confirm the inhibition activity to CntA.

### Inhibitory IC_50_ determination

From a 25 mM stock solution of compound material dissolved in DMSO, a threefold, 10-step serial dilution was prepared and the final concentration of each compound ranges from 300 μM to 0.015 μM. The assay was carried out as per the “UV-visible enzyme assays” and “Inhibitor screening” methodology above, and each compound was incubated with the protein material for 20 min prior to assaying. For each concentration, four replicates were carried out. IC_50_ values were determined using a built-in “Dose Response Inhibition” – [Inhibitor] *versus* response model in Prism 8 for macOS v8.2.0.

### Inhibitor competition assays

As per the Michaelis–Menton kinetics methodology above, we carried a series of four runs in the presence of (i) 0x (control), (ii) 0.25x, (iii) 1x, and (iv) 4x of the IC_50_ value for each inhibitor compound with four independent replicates for each run and fitted to a nonlinear regression Michaelis–Menten model in Prism 8 for macOS v8.2.0.

### Evaluation of inhibitors in *A. baumannii* cell culture

*A. baumannii* cells were cultured overnight in the M9 minimal media at 37 °C with 10 mM L-carnitine as the sole carbon source. The cells were then collected by centrifugation and washed twice in a buffer containing 10 mM Hepes, 1 mM NaCl (pH 7.6). After wash, the cells were resuspended in the same buffer to a density of OD_600_ of 0.4. The cells were aliquoted into 5 ml per replicate, and carnitine was added to a final concentration of 1 mM with/without the addition of the inhibitors (*i.e.*, MMV1, MMV3, and MMV12) at a concentration of 200 μM. A corresponding DMSO-only control was also included. These cells with appropriate treatments were incubated at 37 °C for 1 h. The supernatant was then obtained by filtering through a 0.2-μm filter and analyzed by ion chromatography by quantifying TMA production and carnitine degradation as described previously (13).

### Chemical structures and protein structure depictions

MarvinSketch was used for drawing, displaying, and characterizing chemical structures (Marvin v19.10.0 for Mac, 2019, ChemAxon http://www.chemaxon.com). Reaction summaries were drawn in ChemDraw V17.0 for Mac. Molecular graphics and analyses were performed with UCSF Chimera (66), Chimera X (67), and Coot (59). Protein–ligand interaction maps were generated in Maestro (Academic) v11.9.010 Release 2019-1, Schrodinger.

### *In silico* docking

Structures were prepared in MarvinSketch, saved as .sdf files, and imported into Flare (Cresset) (v3.0). The CntA + MMV020670 pdb file was imported into Flare and minimized using the standard built-in protocols. The ligands were docked using the built-in docking engine using the MMV020670 ligand pose as a template.

## Data availability

The structures of CntA and its ligand-bound forms have been submitted to the PDB database (accession numbers 6Y8J, 6Y8S, 6Y9D, and 6ZGP). All other data have been included in the article.

## Conflict of interest

The authors declare that they have no conflicts of interest with the contents of this article.
