# Supplementary material for: Structural basis of carnitine monooxygenase CntA substrate specificity, inhibition, and intersubunit electron transfer
Source: J Biol Chem. 2020 Nov 23;296:100038. doi: 10.1074/jbc.RA120.016019 (PMC7948474; doi:10.1074/jbc.RA120.016019)
Supplement: Supplementary Figures and Tables [file mmc1.pdf]

Supplementary tables and figures

Table S1. Summary of CntA structural assignments

**Structure**	**Assigned residue range**	**Mononuclear Fe present?**	**[2Fe-2S] present?**	**Oligomeric state**	**RMSD to Apo**
Apo	4-210, 223-240, 256-371	No	Yes	Trimer	-
carnitine-bound	4-239, 256-371	Yes	Yes	Trimer	0.595
gBB-bound	4-214, 224-240, 256-371	Yes	Yes	Trimer	0.379
MMV12-bound	3-247, 253-371	Yes	Yes	Trimer	1.127

gBB = γ-butyrobetaine. MMV12 (MMV020670), an inhibitor compound for CntA.

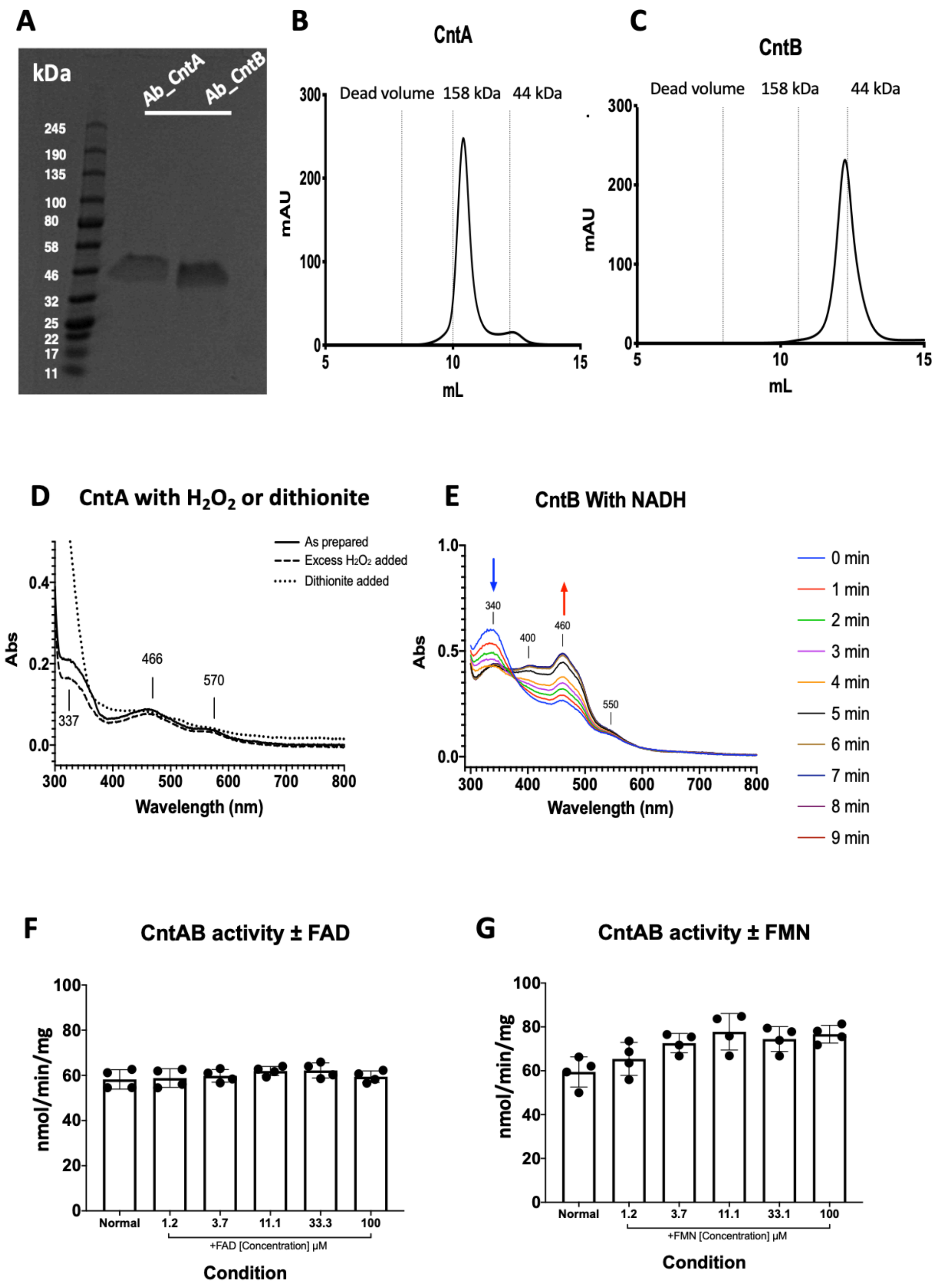

Figure S1 A an SDS-PAGE gel of the purified CntA and CntB proteins. Analytical gel filtrations of CntA (**B**) and CntB (**C**) respectively; **D**, CntA UV-Vis spectrum with added H_2O_2 or dithionate. **E**, CntB UV-Vis spectrum recorded over time showing rapid NADH consumption by the CntB reductase subunit. The addition of FAD (**F**) or FMN (**G**) ranging from 1.2 to 100 µM has little impact on the activity of carnitine oxygenase.

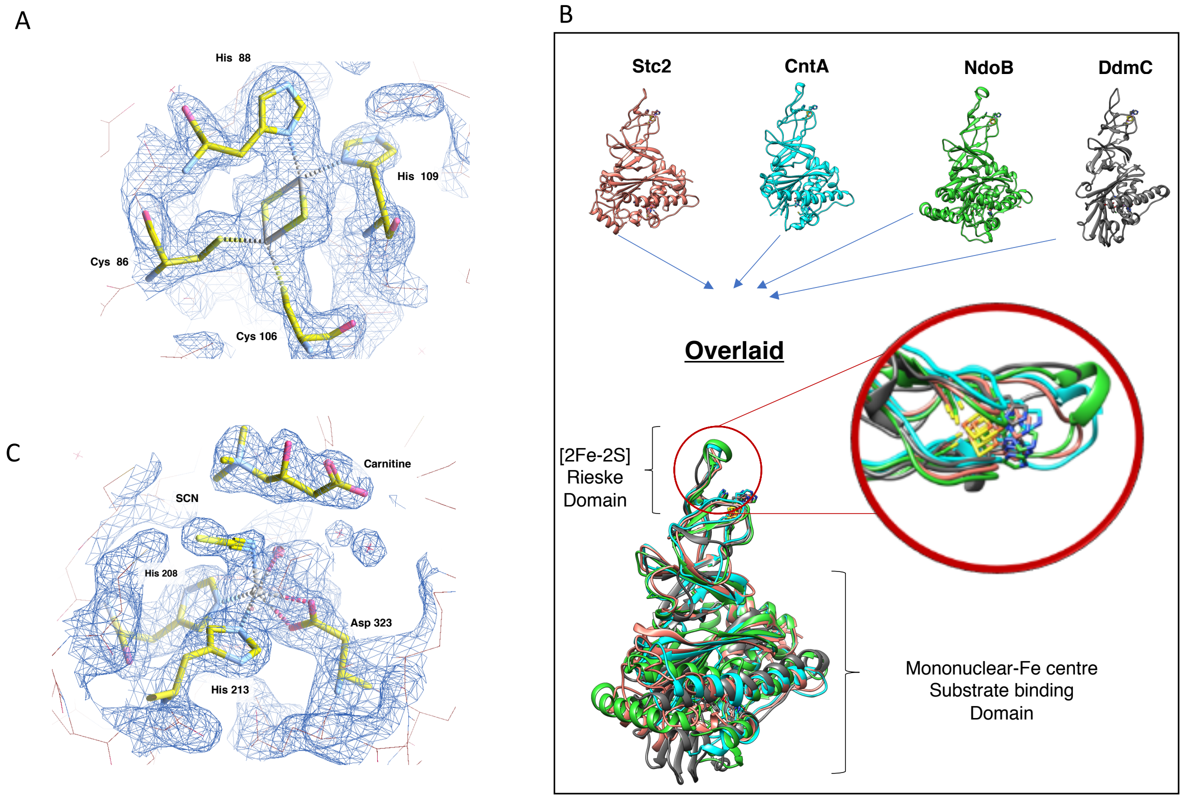

Figure S2 A: $2mF_o - DF_c$ map at 1.5σ of the electron density map (Blue) of the Rieske center in CntA. **B**: Comparison of Rieske oxygenase subunits of Stc2, CntA, NdoB and DmdC with a conserved structural feature in the Rieske domain but high variability in the mononuclear Fe centre. **C**: $2mF_o - DF_c$ map at 1.5σ of the electron density map (Blue) of the Mononuclear Fe centre in CntA coordinated by the His-His-Asp triade of residues, with the Fe also coordinating a water molecule and $[SCN]^-$ ion. The carnitine substrate is shown above.

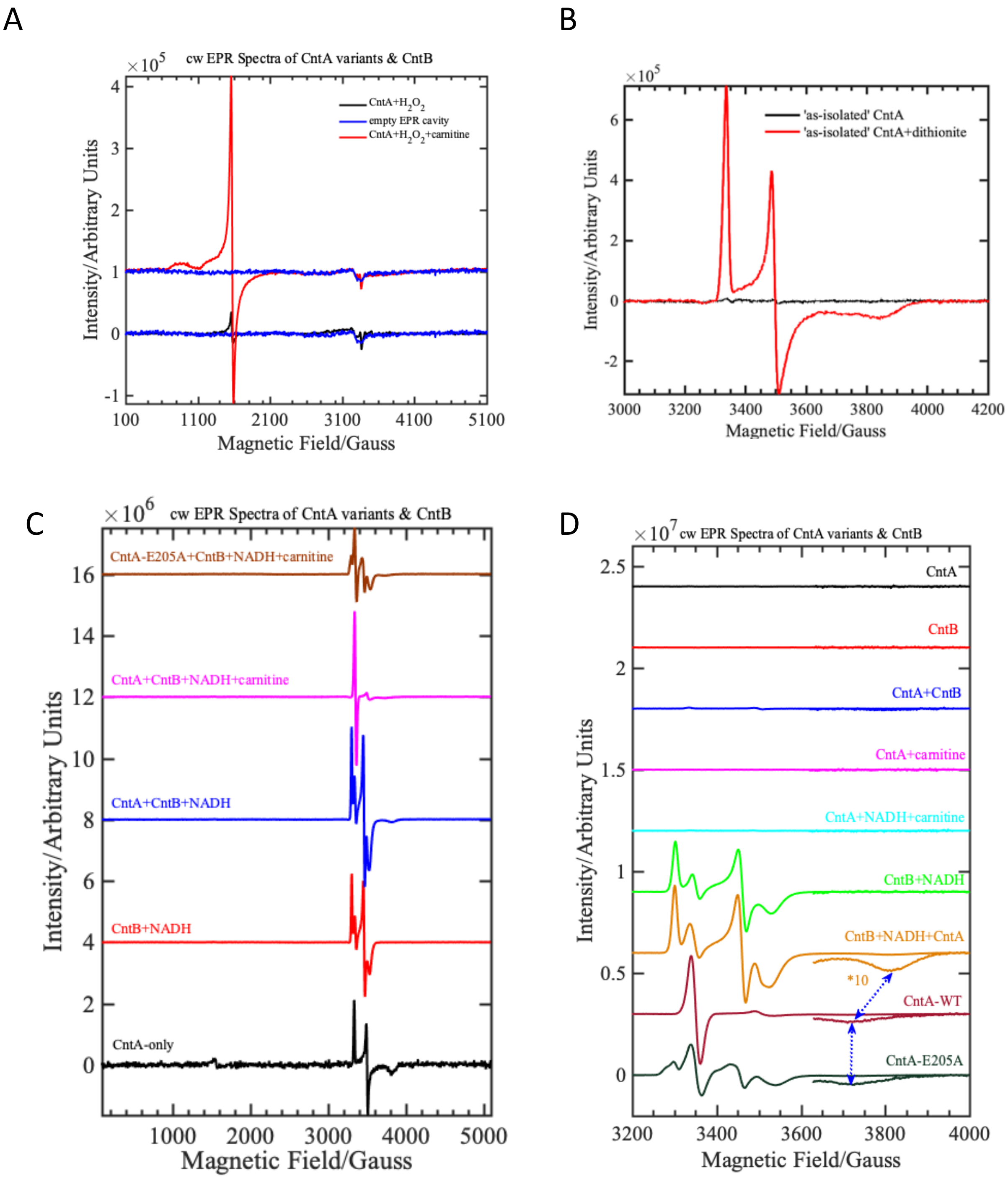

Figure S3 A, The addition of H_2O_2 to the 'as-isolated' CntA in the presence of carnitine giving a strong EPR signal of the high-spin (S = 5/2) ferric iron (red trace) compared to CntA + H_2O_2 only (black trace) and the control (blue trace). **B**, The addition of dithionite to the 'as-isolated' CntA led to the formation of EPR active, one-electron reduced, Rieske $[2Fe\text{-}2S]^{+1}$ centre in CntA, confirming that the Rieske centre present in an oxidised state, $[2Fe\text{-}2S]^{+2}$ in the 'as-isolated' CntA. Wide (Panel **C**) and narrow (Panel **D**) swept, cw-EPR spectra of the CntAB proteins and CntA- E205A mutant under various experimental conditions (CntA, CntB, CntA + CntB, CntA + carnitine, CntA + NADH + carnitine) in the presence and absence of NADH and substrate, L- carnitine respectively; The vertical and slanting double-headed,

dotted blue arrows in figure S3D imply a shift in *g*3 when substrate, L-carnitine is present in addition to CntB+NADH+CntA for the CntA-WT (wide red trace) and CntA-E205A (olive green trace) proteins. The EPR spectrum of the CntA-E205A mutant (Figure S3D; olive green trace) demonstrates the disruption of the intra/inter subunit electron transfer between the reductase (CntB) and oxygenase (CntA) domains. Experimental conditions – as described in Figure 1*F* in main text.

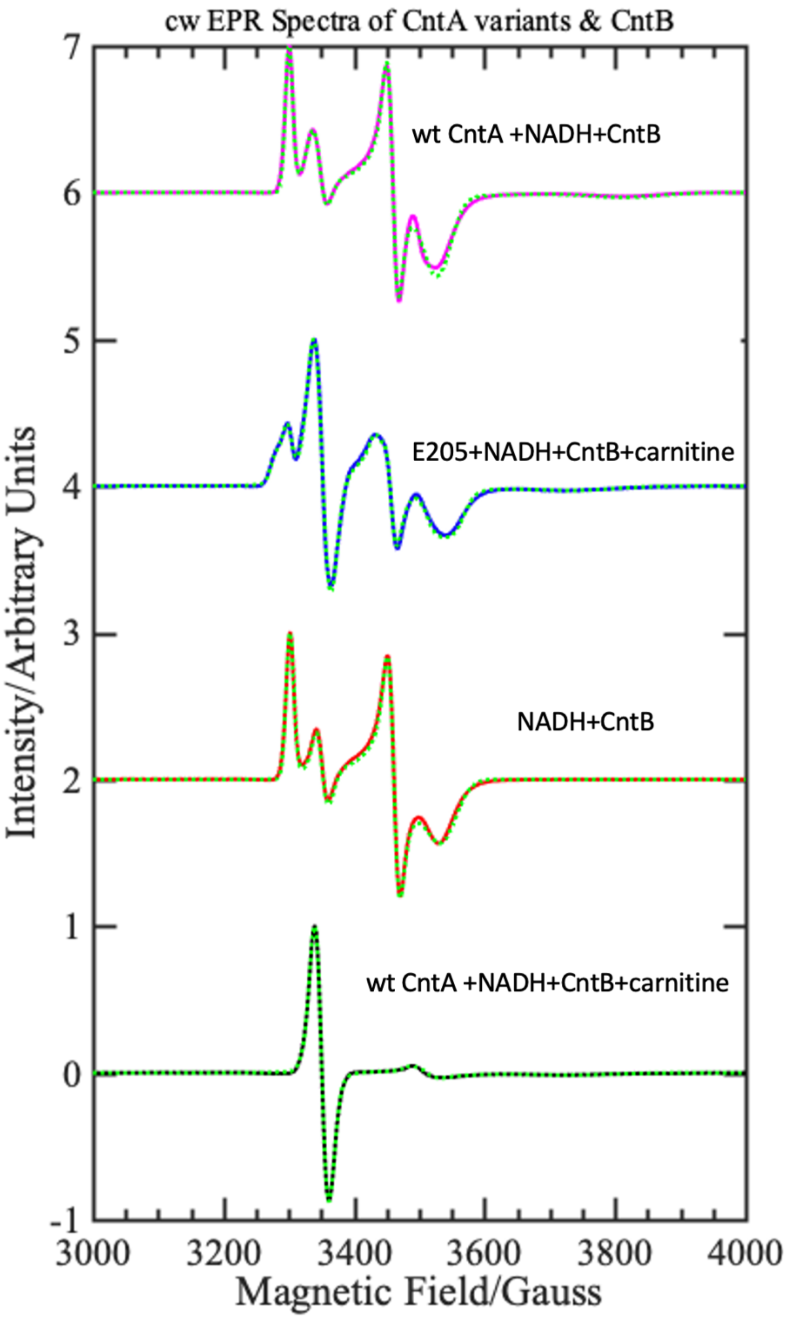

Figure S4, Experimental (black, red, blue and magenta traces) and simulated (green dotted lines) EPR spectra of the CntAB proteins and its mutant, CntA-E205A (blue trace) measured as a frozen solution at 20 K. The EPR spectra were successfully simulated using the spin-Hamiltonian parameters given in **Table 2** of main text; Experimental conditions – as described in Figure 1*F* in main text.

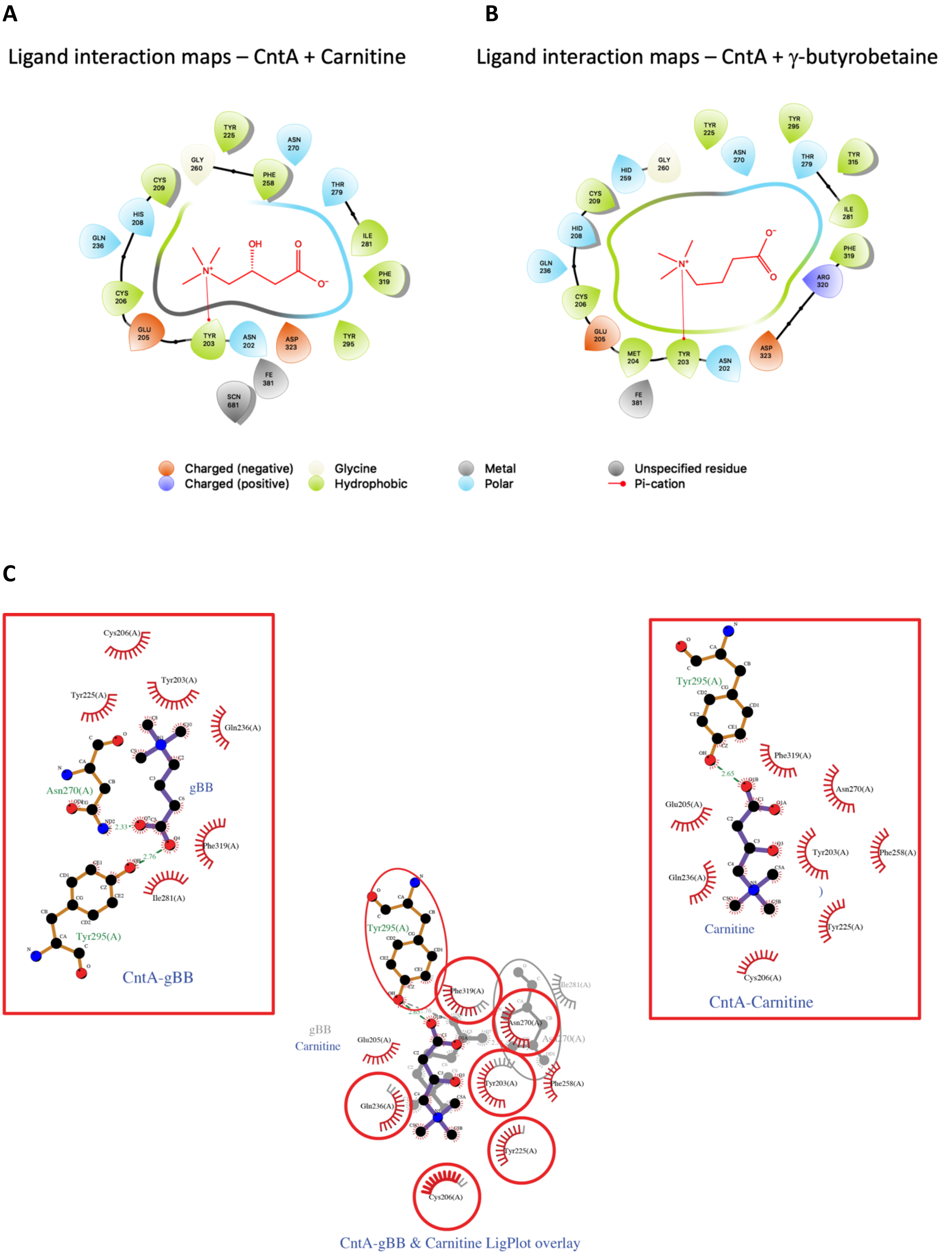

Figure S5 Ligand interaction map of (**A**) carnitine and (**B**) γ-butyrobetaine in the CntA active site. Panel (**C**) shows a LigPlot interaction map with hydrophobic interactions shown as Red eyelashes and hydrogen bonds shown as Green dashed lines. (gBB = γ-butyrobetaine)

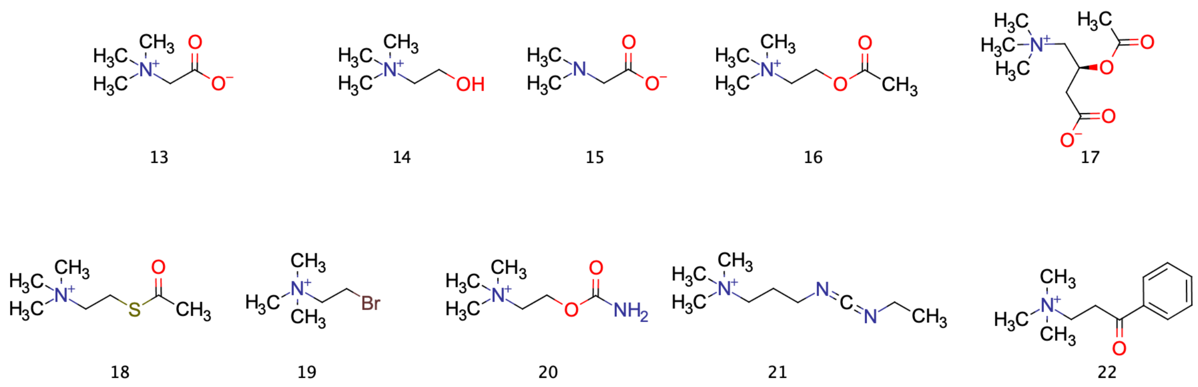

Figure S6 Series of compounds tested for activity: (**13**) Glycine betaine, (14) Choline, (**15**) N,N-dimethylglycine, (**16**) L-acetylcarnitine, (**17**) Acetylcholine, (**18**) Acetylthiocholine, (**19**) (2-Bromoethyl)Trimethylammonium, (**20**) Carbachol, (**21**) 3-{[(Ethylimino)methylene]amino}-N,N,N-trimethyl-1-propanaminium, (**22**) N,N,N-Trimethyl-3-oxo-3-phenylpropan-1-aminium.

A

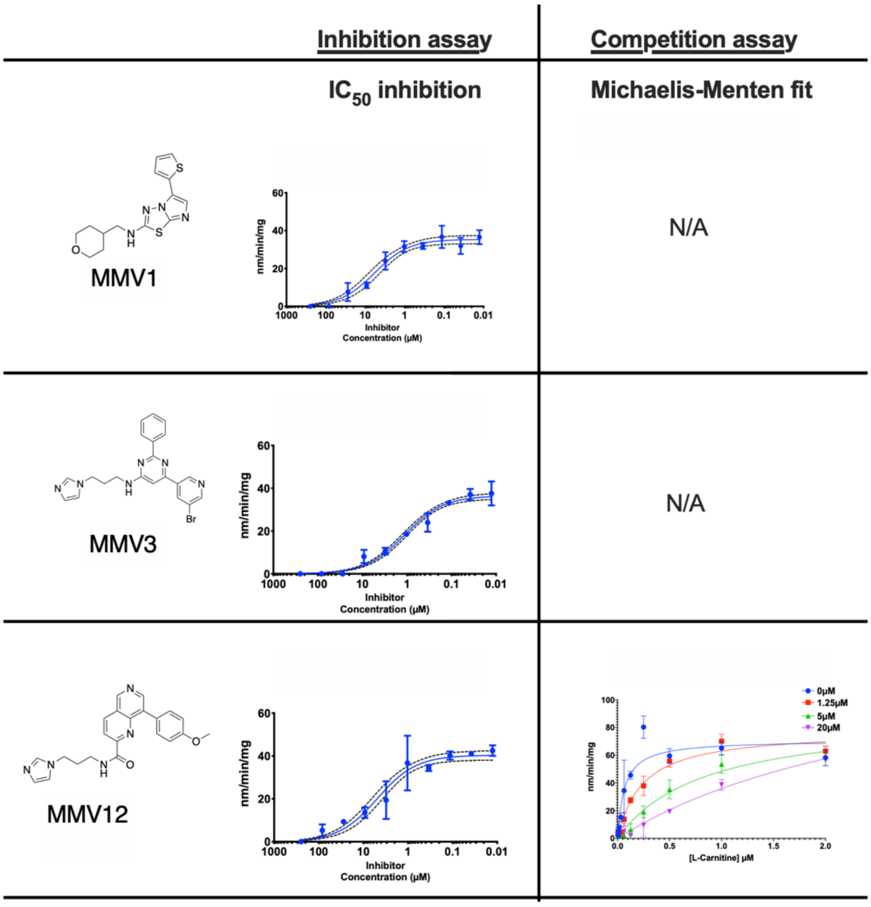

B

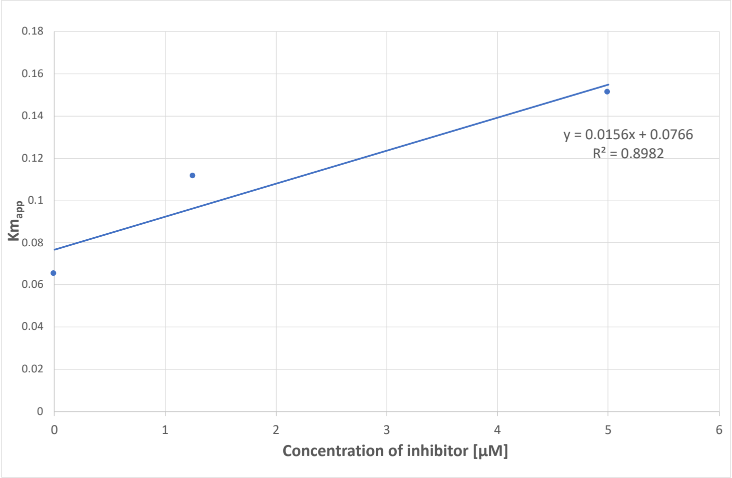

Figure S7, **A:** Assay of inhibition of CntA by the inhibitors. Inhibitory IC_{50} dose responses (column 1) and competition assays for active site competitiveness determination with raw data (column 2) then fitted to a Prism model for competitive inhibitors (column 3). **B:** Plot of Km_{app} vs Inhibitor concentration for MMV12 showing a linear trend at concentrations (0, 1.25 and 5 μM).

A

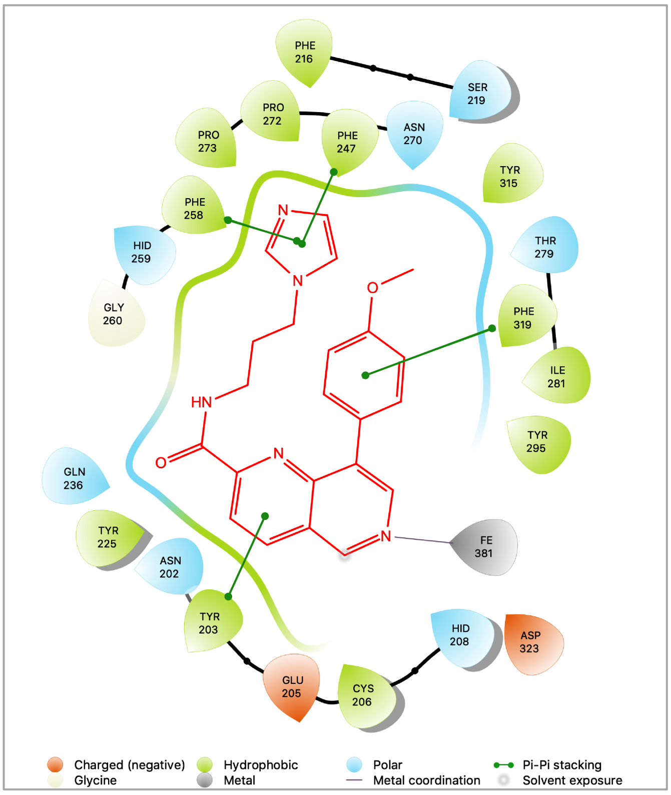

B

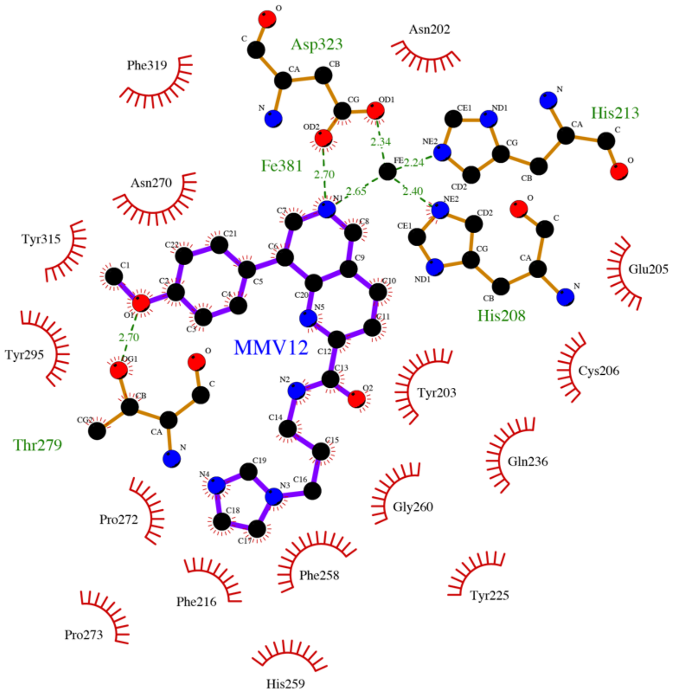

Figure S8 In Panel (**A**) is a ligand interaction map of MMV12 (MMV020670) in the CntA active site and the corresponding LigPlot interaction map in (**B**). Hydrophobic interactions shown as Red eyelashes and hydrogen bonds shown as Red dashed lines.

MMV12

MMV1

MMV3

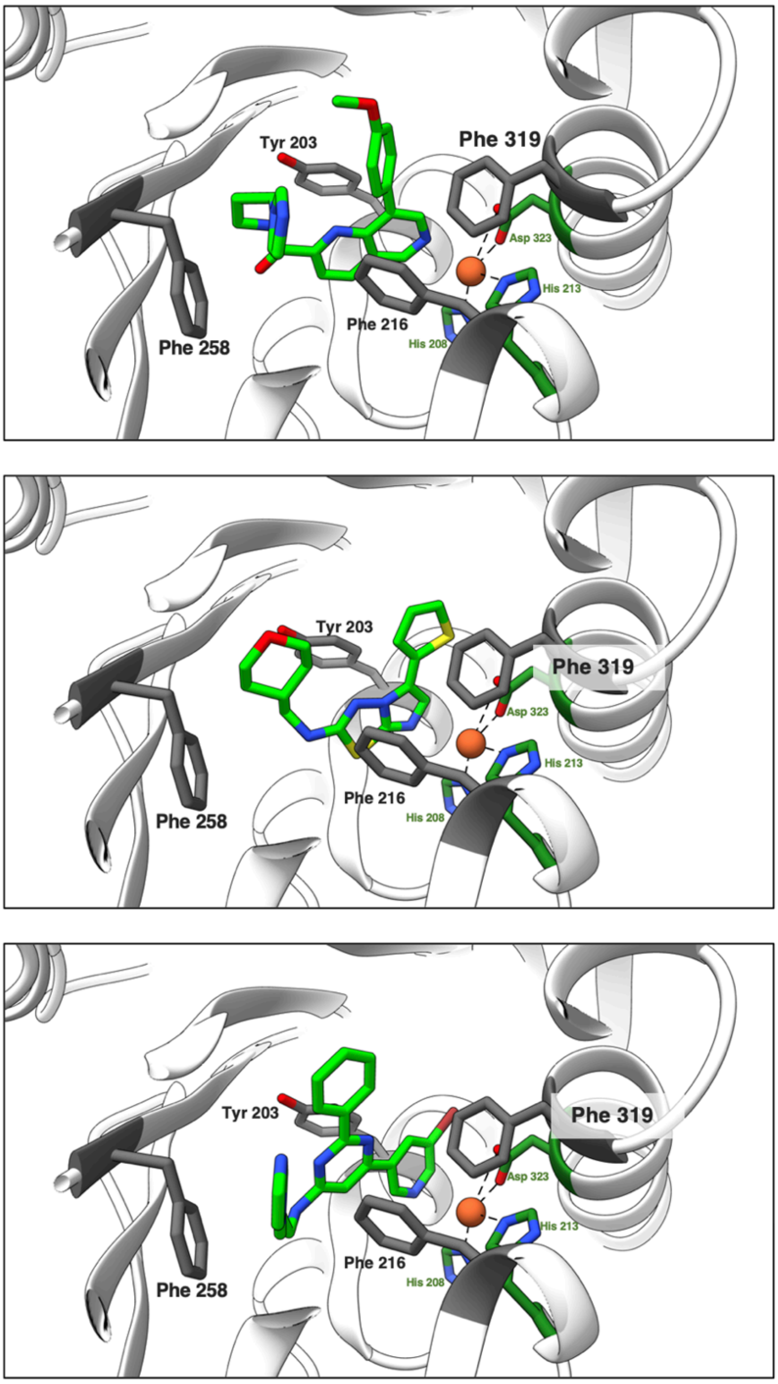

Figure S9, Compound-binding pockets of the inhibitor MMV12 in CntA structure and the docking of MMV1 and MMV3 in CntA.

A

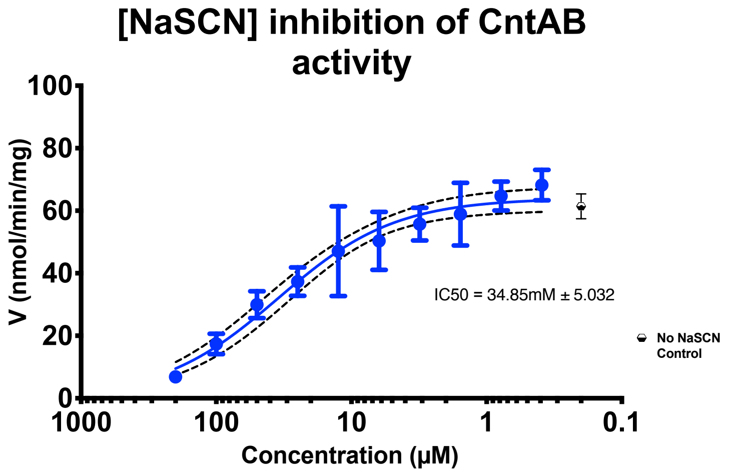

B

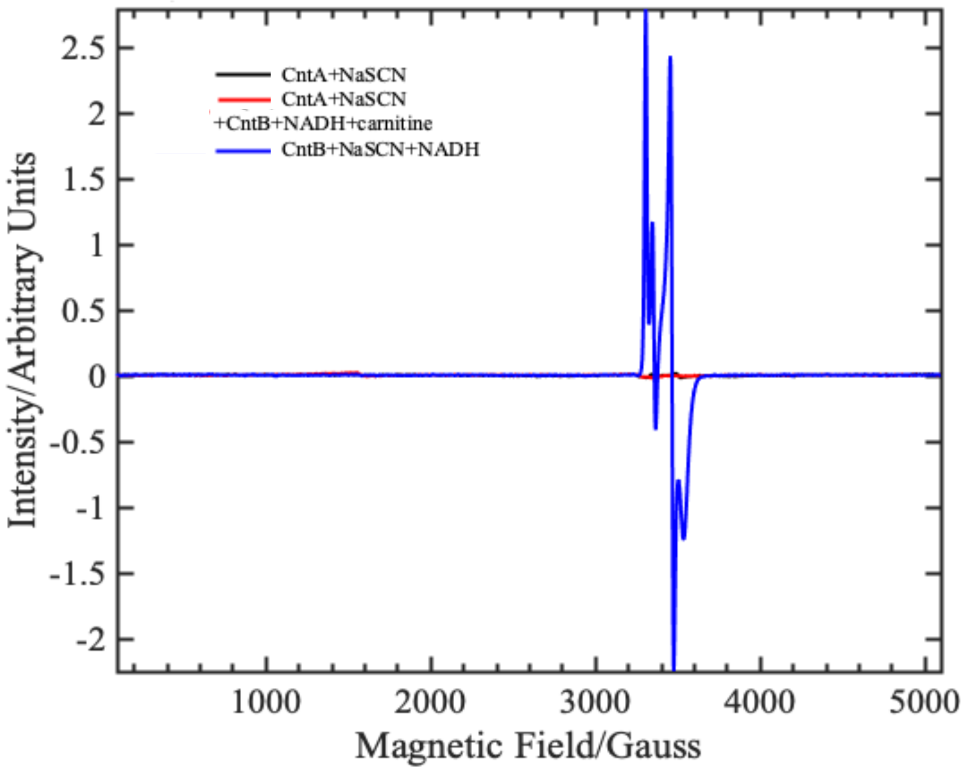

Figure S10 A: Dose response inhibition of CntA activity in the presence of NaSCN (N=3). Error bars are standard deviations and the IC_{50} was calculated in Prism. **B**: cw-EPR spectra of CntA+NaSCN (black trace) and CntA + NaSCN + CntB + NADH + Carnitine (red trace) measured as a frozen solution at 20 K. The observation of EPR silent behaviour for [CntA + NaSCN + CntB + NADH + Carnitine; red trace] implies that NaSCN may have inhibited CntA activity. However, the EPR spectrum of the corresponding control, [CntB + NaSCN + NADH; blue trace], shows the formation of the expected one-electron, reduced ferredoxin, $[2Fe\text{-}2S]^{+1}$ centre and flavin radical demonstrating that the NaSCN does not impact CntB functionality.

Appendix 1

Phylogeny Abbreviations, their corresponding species, and gi/accession numbers in the GenBank database are as follows:

OxoO, *P. putida* 86, 2072732; CarAa, *Pseudomonas* sp. strain CA10, 2317678; VanA, *Pseudomonas* sp. HR199, 1946284; DdmC, *Pseudomonas maltophilia* DI-6, 55584974; TsaM, *Comamonas testosteroni* T-2, 1790867; PobA, *P. pseudoalcaligenes* POB310, 473250; LigX, *Sphingomonas paucimobilis* SYK-6, 4062861; NdmA, *P. putida* CBB5, AFD03116; NdmB, *P. putida* CBB5, AFD03117; BphA1, *P. pseudoalcaligenes* KF707, 151091; IpBAa, *P. putida* RE204, 2822265; BpdC1, *Rhodococcus* sp. strain M5, 927232; BedC1, *P. putida* ML2, 1168640; HcaE, *E. coli* K-12, 81170783; BphA1, *Rhodococcus erythropolis* TA421, 3059209; PhnA1a, *Sphingomonas* sp. strain CHY-1, 75455648; ArhA1, *Sphingomonas* sp. strain A4, 50725019; NahAc, *Pseudomonas* sp. strain JS42, 1224114; DntAc, *Burkholderia cepacia* R34, 17942397; PhtAa, *Mycobacterium vanbaalenii* PYR-1, 49072886; NarAa, *Rhodococcus* sp. strain NCIMB12038, 4704462; NidA, *M. vanbaalenii* PYR-1, 11038552; PhdA, *Nocardioides* sp. strain KP7, 7619816; NidA3, *M. vanbaalenii* PYR-1, 68053509; CmtAb, *P. putida* F1, 1263180; PsbAb, *Rhodopseudomonas palustris*, 5360700; AtdA, *Acinetobacter* sp. strain YAA, 1395141; TdnA1, *P. putida* UCC22, 1841362; AntA, *Acinetobacter* sp. ADP1, 3511232; CbdC, *Burkholderia cepacia* 2CBS, 758210; XylX, *P. putida* TOL, 139861; CmoS, *Spinacia oleracea*, AAB52509; CmoB, *Beta vulgaris*, AAB80954; CmoA, *Amaranthus tricolor*, AAK82768; CntA, *A. baumannii* ATCC 19606, EEX03955; OxyBAC, *Pseudomonas* sp. BIOMIG1, 1057213843; Stc2, *S. meliloti* 1021, 16262853; BmoA, *C. salexigens* DSM 3043, 759867680; GbcA, *P. aeruginosa* PAO1, 15600603.
